# Diagnostic Category Prevalence in 3 Classification Systems Across the Transition to the *International Classification of Diseases, Tenth Revision, Clinical Modification*

**DOI:** 10.1001/jamanetworkopen.2020.2280

**Published:** 2020-04-08

**Authors:** Randall P. Ellis, Heather E. Hsu, Chenlu Song, Tzu-Chun Kuo, Bruno Martins, Jeffrey J. Siracuse, Ying Liu, Arlene S. Ash

**Affiliations:** 1 Department of Economics, Boston University, Boston, Massachusetts; 2 Department of Pediatrics, Boston University School of Medicine, Boston, Massachusetts; 3 BMC HealthNet Plan, Boston, Massachusetts; 4 Analysis Group, Boston, Massachusetts; 5 Division of Vascular and Endovascular Surgery, Boston Medical Center, Boston University School of Medicine, Boston, Massachusetts; 6 Department of Population and Quantitative Health Sciences, University of Massachusetts Medical School, Worcester

## Abstract

**Importance:**

On October 1, 2015, the US transitioned to the *International Classification of Diseases, Tenth Revision, Clinical Modification* (*ICD-10-CM*) for recording diagnoses, symptoms, and procedures. It is unknown whether this transition was associated with changes in diagnostic category prevalence based on diagnosis classification systems commonly used for payment and quality reporting.

**Objective:**

To assess changes in diagnostic category prevalence associated with the *ICD-10-CM* transition.

**Design, Setting, and Participants:**

This interrupted time series analysis and cross-sectional study examined level and trend changes in diagnostic category prevalence associated with the *ICD-10-CM* transition and clinically reviewed a subset of diagnostic categories with changes of 20% or more. Data included insurance claim diagnoses from the IBM MarketScan Commercial Database from January 1, 2010, to December 31, 2017, for more than 18 million people aged 0 to 64 years with private insurance. Diagnoses were mapped using 3 common diagnostic classification systems: World Health Organization (WHO) disease chapters, Department of Health and Human Services Hierarchical Condition Categories (HHS-HCCs), and Agency for Healthcare Research and Quality Clinical Classification System (AHRQ-CCS). Data were analyzed from December 1, 2018, to January 21, 2020.

**Exposures:**

US implementation of *ICD-10-CM*.

**Main Outcomes and Measures:**

Monthly rates of individuals with at least 1 diagnosis in a diagnostic classification category per 10 000 eligible members.

**Results:**

The analytic sample contained information on 2.1 billion enrollee person-months with 3.4 billion clinically assigned diagnoses; the mean (range) monthly sample size was 22.1 (18.4 to 27.1 ) million individuals. While diagnostic category prevalence changed minimally for WHO disease chapters, the *ICD-10-CM* transition was associated with level changes of 20% or more among 20 of 127 HHS-HCCs (15.7%) and 46 of 282 AHRQ-CCS categories (16.3%) and with trend changes of 20% or more among 12 of 127 of HHS-HCCs (9.4%) and 27 of 282 of AHRQ-CCS categories (9.6%). For HHS-HCCs, monthly rates of individuals with any acute myocardial infarction diagnosis increased 131.5% (95% CI, 124.1% to 138.8%), primarily because HHS added non–ST-segment–elevation myocardial infarction diagnoses to this category. The HHS-HCC for diabetes with chronic complications increased by 92.4% (95% CI, 84.2% to 100.5%), primarily from including new diabetes-related hypoglycemia and hyperglycemia codes, and the rate for completed pregnancy with complications decreased by 54.5% (95% CI, –58.7% to –50.2%) partly due to removing vaginal birth after cesarean delivery as a complication.

**Conclusions and Relevance:**

These findings suggest that the *ICD-10-CM* transition was associated with large prevalence changes for many diagnostic categories. Diagnostic classification systems developed using *ICD-9-CM* may need to be refined using *ICD-10-CM* data to avoid unintended consequences for disease surveillance, performance assessment, and risk-adjusted payments.

## Key Points

**Question:**

Was the transition from *International Classification of Diseases, Ninth Revision, Clinical Modification* (*ICD-9-CM*) to the *Tenth Revision* (*ICD-10-CM*) in October 2015 associated with changes in diagnostic category prevalence when diagnoses are grouped by classification system?

**Findings:**

This interrupted time series analysis and cross-sectional study examined insurance claims for more than 18 million privately insured adults and children in the US from 2010 to 2017 and found instantaneous increases or decreases of 20% or more associated with the *ICD-10-CM* transition for nearly 1 in 6 (16%) diagnostic categories in 2 of 3 influential diagnostic classification systems.

**Meaning:**

These findings suggest that diagnostic classification systems developed with *ICD-9-CM* data may need to be refined for use with *ICD-10-CM* data for disease surveillance, performance assessment, or risk-adjusted payment.

## Teaser

This interrupted time series analysis and cross-sectional study examines changes in the prevalence of diagnostic codes before the implementation of the *International Classification of Diseases, Tenth Revision, Clinical Modification* (*ICD-10-CM*) vs after.

## Introduction

Diagnostic codes are widely used within health care in the US for reimbursement, quality assessment, public health reporting, calculating risk-adjusted payments, and studying clinical outcomes. In October 2015, the US switched its diagnostic coding system from the *International Classification of Diseases, Ninth Revision, Clinical Modification* (*ICD-9-CM*) to the more detailed *International Statistical Classification of Diseases, Tenth Revision, Clinical Modification (ICD-10-CM)*, expanding the number of available codes nearly 5-fold.^1^ Previous studies have noted discontinuities in specific disease or population subgroups.^2,3,4,5,6,7,8^ This interrupted time series analysis and cross-sectional study sought to quantify the magnitude of change in prevalence in all diagnostic categories for 3 widely used diagnostic classification systems and to explore potential reasons for these changes through selected clinical review.

## Methods

### Data and Study Sample

We used the IBM MarketScan Commercial Database from January 1, 2010, to December 31, 2017, including individuals ages 0 to 64 years enrolled in noncapitated commercial insurance plans with both medical and pharmacy benefits. The MarketScan database contains deidentified health care claims information from US employers, health plans, and hospitals. Data on all enrollment records and inpatient, outpatient, ancillary, and drug claims are collated and linked to individuals. The age and sex distribution of the eligible population varied little over the 96 months studied. Boston University’s institutional review board exempted this study from review and informed consent owing to the use of deidentified data. Data were analyzed from December 1, 2018, to January 21, 2020.

### Outcome

Our primary outcome was the monthly rate of individuals with at least 1 diagnosis in a diagnostic classification category per 10 000 enrollees. We used claim dates of service, health care practitioner type, and inpatient and outpatient procedure codes to identify claims that are allowed to contribute diagnostic codes, mimicking Department of Health and Human Services (HHS) filters used in risk adjustment, which only consider diagnoses from an acceptable health care practitioner claim type: hospital inpatient, hospital outpatient, or professional.^9^ Monthly prevalence rates were examined for changes in reported diagnostic category prevalence at and following the October 2015 *ICD-10-CM* transition.

### Diagnostic Classification Systems

There were 14 567 allowable *ICD-9-CM* codes in 2015 vs 71 486 allowable *ICD-10-CM* codes in 2017 to describe patient medical conditions.^10^ To reduce complexity, predictive models and disease tracking systems rely on classification systems (ie, groupers) to collapse these codes into a smaller number of broadly homogeneous clinical categories. We explored 3 commonly used classification systems.

The World Health Organization (WHO) maps codes into chapters, which are commonly used for epidemiology and surveillance, based on their first 3 alphanumeric characters. The WHO’s *ICD-9-CM* mapping had 19 chapters, which expanded to 21 chapters with the transition to *ICD-10-CM* by separating previously grouped eye, ear, and neurological disorders into distinct chapters. To facilitate cross-year comparisons, we maintained the 19-chapter structure.^11^

The HHS Hierarchical Condition Categories (HHS-HCCs) system was developed for the Patient Protection and Affordable Care Act (ACA) marketplace risk adjustment payment model and is used to allocate funds among competing health plans.^12^ The HHS-HCC system used 127 condition categories for the ACA population when it was implemented from 2014 through 2017, and its mappings were updated to accommodate *ICD-10-CM* diagnoses in 2015.^13^ To ensure that recently added diagnostic codes were recognized, we applied the 2017 HHS-HCC software to the full range of study data for 2010 to 2017. For sensitivity analysis, we evaluated the effects of the HHS filtering logic and the 2015 HHS software with the original *ICD-10-CM* mappings.

The Agency for Healthcare Research and Quality Clinical Classification System (AHRQ-CCS) categories are used by managed care plans, insurers, researchers, and surveillance systems for payment, quality assessment, and epidemiology. Its categories are also widely used for risk adjustment and examining disease trends.^14,15^ The AHRQ-CCS largely maintained its category structure with the transition to *ICD-10-CM*, and we used its 282 diagnostic categories appearing in our data before and after the transition applying the 2017 category mappings.

All 3 classification systems updated their diagnostic categories to accommodate *ICD-10-CM*.^16,17,18^ However, because only 5% of *ICD-9-CM* codes can be mapped 1-to-1 to *ICD-10-CM*^19^ and the crosswalk algorithms were developed before *ICD-10-CM* coding was adopted for clinical care, it was not possible to prospectively examine the continuity of diagnostic categories across the transition. For comparability across classification systems, we generated the condition categories described by Kautter et al^12^ but did not impose the hierarchies used in the HHS-HCC model.

### Statistical Analysis

To derive the monthly diagnostic category prevalence rates, we first applied HHS software filters^9^ to calendar months based on the earliest service date of each claim line. We then standardized monthly disease prevalence to eliminate variation based on the number of days in the month. In each month, we counted the number of distinct individuals with any diagnosis code in a category, divided by the number of people enrolled in that month, and multiplied by 30.437 (the mean number of days in a month) per day in the month. This upweights prevalence rates in short months, such as February and September, by between 1.5% and 8.7% and downweights 31-day months, such as October, by 1.8%. We also examined trends in available population descriptors: sample size, age, sex, preferred provider organization and health maintenance organization enrollment, and fraction in California (the state with the largest representation in our sample). This confirmed that no large changes in the study population occurred during our study period (eFigure 1 in the Supplement). Therefore, we conducted all analyses without controlling for these variables.

We used piecewise linear regression models to examine level and trend changes in standardized monthly diagnostic category prevalence associated with the October 2015 *ICD-10-CM* transition for each diagnostic category in all 3 classification systems. All regressions included time (to model secular trends), a post–*ICD-10-CM* indicator variable to estimate any immediate level change associated with the *ICD-10-CM* transition, a 2-way interaction term to determine whether the *ICD-10-CM* transition was associated with a change in trend, and dummy variables for each month to control for seasonality (eAppendix in the Supplement).

We conducted statistical significance tests for all diagnostic categories in each classification system. However, in this article, we focus attention on 3 common conditions: diabetes, cardiac disease, and pregnancy. Within each condition we selected 3 HHS-HCC categories that illustrate a range of observed patterns and chose clinically similar WHO chapters and AHRQ-CCS categories for comparison.

#### Statistical Tests

We used 2-sided *t* tests to identify statistically significant changes in level or trend in regression results and *F* tests to look for the joint significance of level and trend changes. We performed 2 *F* tests for each diagnostic category: one to test the hypothesis of a straight line with no change in October 2015 (ie, test of linearity), and the other to test whether the predicted diagnostic category prevalence in December 2017 differed from a prediction based on extending the straight line fit to pre–*ICD-10-CM* data (ie, cumulative effect of changes in level and time trend).

For all tests, we applied the Bonferroni multiple-testing correction^20^ so that *P* values were considered statistically significant only when they were less than .05 divided by the number of categories in the classification system. For example, in HHS-HCC regressions, statistical significance required *P* < .0004 (ie, .05/127) for statistical significance in *F* tests. We considered changes of 20% or more to be large.

Each monthly rate was calculated on more than 18 million individuals, making them extremely precise. However, each statistical test examined changes in time using 96 observations. A significant finding indicates that inclusion of a level or slope change at October 2015 describes the temporal pattern in the observed data better than a straight line.

#### Data Interpretation and Visualization

To facilitate meaningful comparisons of changes across condition categories with vastly different mean prevalence, we normalized all findings by dividing rates by the mean diagnostic category prevalence from September 2015, the month prior to the *ICD-10-CM* transition. To visualize patterns of observed changes, we created time series graphs for all diagnostic categories in each classification system. Each graph depicts 3 normalized series: (1) observed diagnostic category prevalence, (2) piecewise linear model predicted prevalence, and (3) locally estimated scatterplot smoothing curves, fit separately to the pretransition and posttransition periods. This last series helps to visualize possible nonlinear trends. While raw (ie, nonnormalized) rates were used for regression modeling and testing, we describe these changes as percentages to focus on their size relative to the base rate in September 2015.

#### Clinical Review

We used clinical review to explore potential reasons for large changes at the time of transition from *ICD-9-CM* to *ICD-10-CM* for diabetes-, cardiac-, and pregnancy-related conditions in the examined classification systems. For each HHS-HCC and AHRQ-CCS condition category with a statistically significant change of 20% or more in prevalence level or trend that was selected for clinical review, we examined frequencies of individual *ICD-9-CM* and *ICD-10-CM* diagnoses before and after the *ICD-10-CM* transition. We also reviewed guidance documents for hospital coding staff regarding changes in coding practices related to the *ICD-10-CM* transition for these diagnostic categories.

## Results

The analytic sample contained information on 2.1 billion enrollee person-months with 3.4 billion clinically assigned diagnoses; the mean (range) monthly sample size was 22.1 (18.4 to 27.1) million individuals. The transition from *ICD-9-CM* was effectively instantaneous, with 99.8% of all diagnoses coded using *ICD-10-CM* in October 2015.

We examined study population characteristics by month from 2010 through 2017 to look for changes that might have introduced trend artifacts (eFigure 1 in the Supplement). Although the number of eligible enrollees varied by year, population characteristics remained nearly constant across all months. In particular, no noteworthy changes in the number of eligible enrollees, mean age, sex, health insurance plan type, or fraction of enrollees located in California occurred at the October 2015 transition to *ICD-10-CM*. The proportion of enrollees with at least 1 eligible diagnosis in a month was consistent over the sample period.

Table 1 summarizes overall findings on changes in diagnostic category prevalence rates within each classification system. No large changes were observed in the WHO chapters, suggesting that overall coding practice (and population health) was relatively stable across the transition. However, the *F* test rejects simple linearity in favor of a model with a discontinuity in slope or trend for 11 of 17 WHO chapters (58%), identifying small changes at the *ICD-10-CM* transition but none that approached the threshold that we considered large (ie, ≥20%) .

**Table 1.** Changes in Monthly Prevalence of Diagnostic Categories After *International Statistical Classification of Diseases, Tenth Revision, Clinical Modification* by Classification System

Change	No. (%)		
	WHO chapters (n = 19)	Categories	
		HHS-HCC (n = 127)	AHRQ-CCS (n = 282)^a^
Large changes^b^			
Level	0	20 (15.7)	46 (16.3)
Trend	0	12 (9.4)	27 (9.6)
Cumulative by			
20% or more	0	30 (23.6)	61 (21.6)
50% or more	0	12 (9.4)	28 (9.9)
*F* test rejected a straight line	11 (58)	74 (58.3)	165 (58.5)

Abbreviations: AHRQ-CCS, Agency for Healthcare Research and Quality Clinical Classification System; HHS-HCC, Department of Health and Human Services Hierarchical Condition Category; WHO, World Health Organization.

^a^ Three AHRQ-CCS categories with 0 cases under *ICD-10-CM* were excluded.

^b^ Large changes were defined as an increase or decrease by 20% or more.

In contrast, changes of 20% or more were common under the HHS-HCC and AHRQ-CCS systems: statistically significant and large changes in level were found for 20 of 127 HHS-HCCs (15.7%) and 46 of 282 AHRQ-CCS categories (16.3%). Large changes in trend occurred in 12 of 127 HHS-HCCs (9.4%) and 27 of 282 AHRQ-CCS categories (9.6%). Given the hundreds of categories in the HHS and AHRQ classification systems, we selected a subset of categories relating to diabetes, cardiac disease, and pregnancy to illustrate some of the largest discontinuities for the HHS-HCC system. Tables summarizing regression results for all diagnostic categories in each classification system are presented in eTable 1 in the Supplement for the WHO chapters, eTable 2 in the Supplement for HHS-HCCs, and eTable 3 in the Supplement for AHRQ-CCS categories.

Table 2 summarizes results for 9 selected HHS-HCCs. Using regressions with 96 monthly observations, we found large level changes in October 2015 for diabetes with chronic complications (92.4% [95% CI, 84.2% to 100.5%]) and without chronic complications (−19.1% [95% CI, −25.3% to −12.9%]), acute myocardial infarction (AMI) (131.5% [95% CI, 124.1% to 138.8%]), unstable angina and other acute ischemic heart disease (−31.6% [95% CI, −37.5% to −25.7%]), and pregnancies with major complications (−44.6% [95% CI, −53.8% to −35.4%]) and with complications (−54.5% [95% CI, −58.7% to −50.2%]). We also found large changes in trend in diabetes with acute complications (24.4% [95% CI, 11.7% to 37.1%]) and chronic complications (23.7% [95% CI, 11.7% to 35.7%]) and in pregnancies with major complications (19.5% [95% CI, 6.0% to 33.0%]). There were large cumulative effects of level and trend changes in 7 of the 9 selected HCCs, with the 3 largest being AMI (147.4% [95% CI, 138.6% to 156.1%]), diabetes with chronic complications (116.1% [95% CI, 106.4% to 125.8%]), and completed pregnancy with complications (−50.8% [95% CI, −55.9% to −45.7%]).

**Table 2.** Changes in Prevalence Levels and Trends Since *ICD-10-CM* Implementation for Selected HHS-HCCs

Condition categories	HHS-HCC code	Prevalence/10 000 enrollees in September 2015 (95% CI)^a^	Change since *ICD-10-CM*, % (95% CI) [*P* value]^a^		
			Level change^b^	Time trend effect^c^	Cumulative effect of level and time trend^d^
Diabetes					
With acute complications	CC19	0.89 (0.84 to 0.94)	2.8 (−5.8 to 11.4) [.26]	24.4 (11.7 to 37.1) [<.001]	27.2 (16.9 to 37.5) [<.001]
With chronic complications	CC20	27.52 (27.25 to 27.79)	92.4 (84.2 to 100.5) [<.001]	23.7 (11.7 to 35.7) [<.001]	116.1 (106.4 to 125.8) [<.001]
Without complication	CC21	116.77 (116.21 to 117.33)	−19.1 (−25.3 to −12.9) [<.001]	−7.6 (−16.7 to 1.4) [.005]	−26.7 (−34.1 to −19.4) [<.001]
Cardiac disease					
Congestive heart failure	CC130	11.82 (11.64 to 12)	4.1 (−1.9 to 10.6) [.02]	−5.6 (−14.9 to 3.6) [.04]	−1.3 (−8.8 to 6.2) [.56]
Acute myocardial infarction	CC131	0.88 (0.83 to 0.93)	131.5 (124.1 to 138.8) [<.001]	15.9 (5.1 to 26.7) [<.001]	147.4 (138.6 to 156.1) [<.001]
Unstable angina and other acute ischemic heart disease	CC132	2.85 (2.76 to 2.94)	−31.6 (−37.5 to −25.7) [<.001]	−7.5 (−16.1 to 1.2) [.004]	−39.1 (−46.1 to −32.0) [<.001]
Completed pregnancy					
With major complications	CC207	0.40 (0.37 to 0.43)	−44.6 (−53.8 to −35.4) [<.001]	19.5 (6.0 to 33.0) [<.001]	−25.1 (−36.1 to −14.1) [<.001]
With complications	CC208	5.17 (5.05 to 5.29)	−54.5 (−58.7 to −50.2) [<.001]	3.7 (−2.6 to 9.9) [.046]	−50.8 (−55.9 to −45.7) [<.001]
With no or minor complications	CC209	12.40 (12.22 to 12.58)	2.8 (−0.2 to 5.9) [.002]	−3.3 (−7.8 to 1.1) [.01]	−0.4 (−4.0 to 3.2) [.67]

Abbreviations: HHS-HCC, Health and Human Services Hierarchical Condition Category; *ICD-10-CM*, *International Statistical Classification of Diseases, Tenth Revision, Clinical Modification*.

^a^ All 95% CIs are adjusted using the Bonferroni correction for multiple tests.

^b^ Calculated as the coefficient on a dummy indicator for implementation of *ICD-10-CM*, normalized by the base rate.

^c^ Calculated based on the post–*ICD-10-CM* indicator interacted with time and shows the change during 26 months after implementation of *ICD-10-CM*.

^d^ Calculated as the level change and the time trend effect over the 26 months since *ICD-10-CM*.

**Figure 1.**
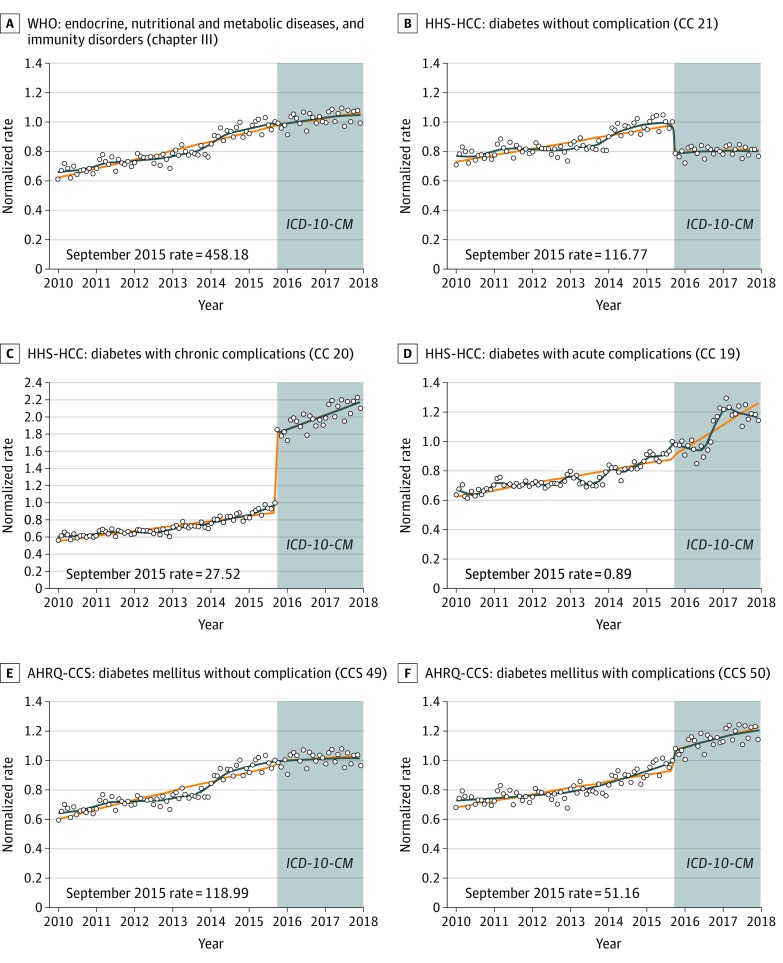
Monthly Diagnostic Category Prevalence Rates of Diabetes Stratified by Classification System The diagnostic category prevalence rate per 10 000 enrollees in September 2015 is presented in the lower left corner of each panel as the reference rate. Curves are fitted with a discontinuity at October 2015. Circles indicate the normalized rate (rate of enrollees with at least 1 diagnosis in a diagnostic category divided by the September 2015 rate); orange lines, predicted rate using a piecewise linear model; blue lines, predicted rate using locally estimated scatterplot smoothing; AHRQ-CCS, Agency for Healthcare Research and Quality Clinical Classification System; HHS-HCC, Health and Human Services Hierarchical Condition Category; *ICD-10-CM*, *International Statistical Classification of Diseases, Tenth Revision, Clinical Modification*; and WHO, World Health Organization.

Figure 1 shows monthly diagnostic category prevalence over time for all diabetes-related categories in all 3 classification systems. Figure 1A reveals that the number of individuals with at least 1 code for an endocrine system diagnosis continued its previous upward trend across the *ICD-10-CM* transition. Figure 1B and C illustrate the immediate level changes in October 2015 for the HHS-HCC system: lower for diabetes without complications and higher for diabetes with chronic complications. There were no large changes in the AHRQ-CCS categories for diabetes with complications or diabetes without complications at the *ICD-10-CM* transition.

Clinical review revealed that level changes in HHS-HCCs were largely explained by new codes in *ICD-10-CM* that explicitly link certain conditions to diabetes. For example, in *ICD-9-CM*, an individual separately coded as having diabetes and hypoglycemia would not necessarily be tagged as having diabetes with complications. However, the HHS-HCC system maps the single *ICD-10-CM* code for diabetes with hypoglycemia to a diabetes complication category, reducing the number of individuals classified as having diabetes without complications.

**Figure 2.**
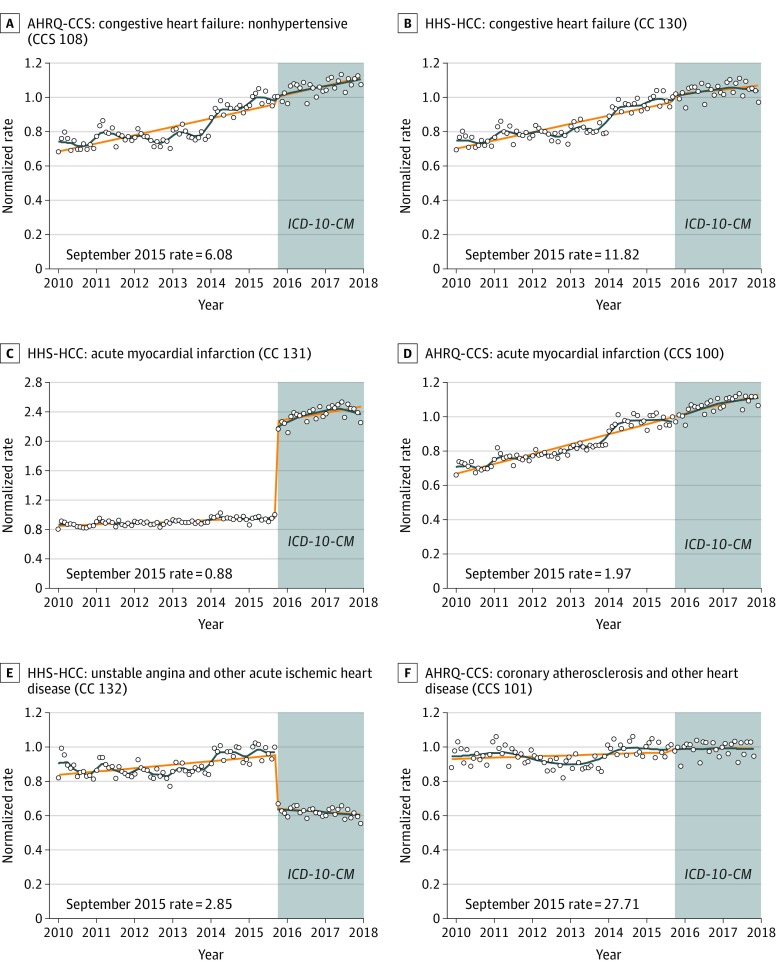
Monthly Diagnostic Category Prevalence Rates of Selected Cardiac Conditions Stratified by Classification System The diagnostic category prevalence rate per 10 000 enrollees in September 2015 is presented in the lower left corner of each panel as the reference rate. Curves are fitted with a discontinuity at October 2015. Circles indicate the normalized rate (rate of enrollees with at least 1 diagnosis in a diagnostic category divided by the September 2015 rate); orange lines, predicted rate using a piecewise linear model; blue lines, predicted rate locally estimated scatterplot smoothing algorithm; AHRQ-CCS, Agency for Healthcare Research and Quality Clinical Classification System; HHS-HCC, Health and Human Services Hierarchical Condition Category; and *ICD-10-CM*, *International Statistical Classification of Diseases, Tenth Revision, Clinical Modification*.

Figure 2 depicts monthly diagnostic category prevalence rates for 6 selected cardiac diagnostic categories. The HHS-HCC system showed a decline in unstable angina and other acute ischemic heart disease and an increase in AMI. In contrast, there were no meaningful changes in AMI or its broader category, coronary atherosclerosis and other heart disease, using the AHRQ-CCS system. Likewise, there were no changes in prevalence among congestive heart failure categories in either the HHS-HCC or AHRQ-CCS systems, or for the broad WHO chapter grouping diseases of the circulatory system (eFigure 2 in the Supplement).

Clinical review found that the differences between trends in AMI within the HHS-HCCs and AHRQ-CCS categories are largely explained by how these systems accommodated the *ICD-10-CM* non–ST-elevation myocardial infarction (non-STEMI) diagnoses: the HHS-HCC system started mapping non-STEMI codes into its AMI category in 2015, while AHRQ-CCS had already done so previously.

**Figure 3.**
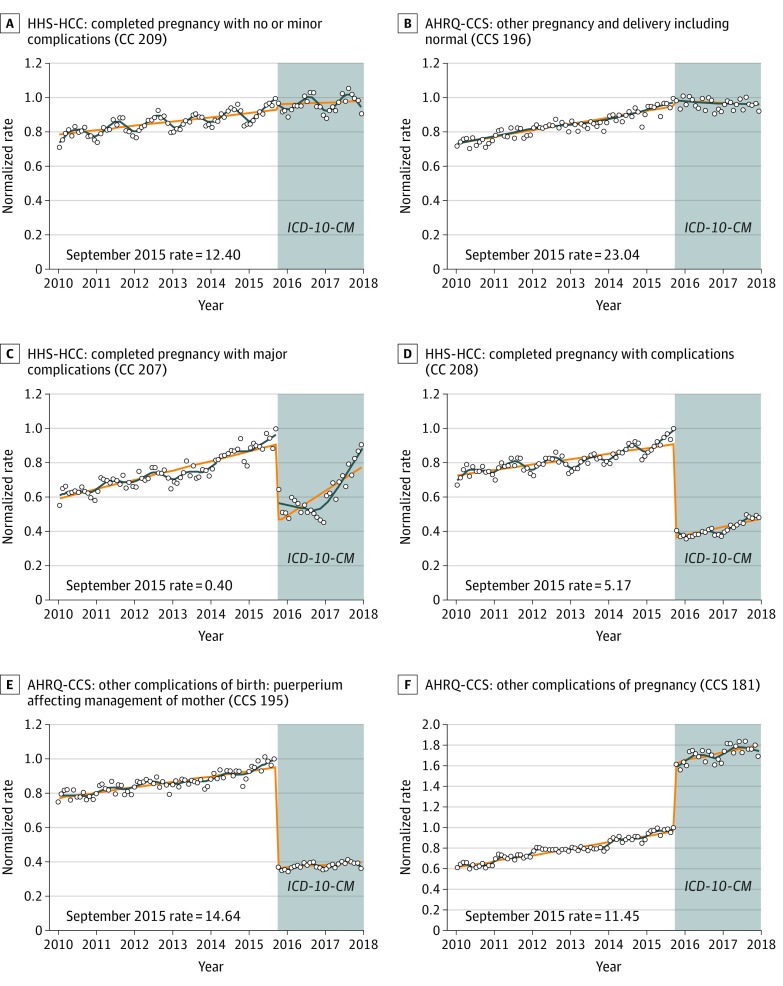
Monthly Diagnostic Category Rates of Selected Pregnancy-Related Conditions Stratified by Classification System The diagnostic category prevalence rate per 10 000 enrollees in September 2015 is presented in the lower left corner of each panel as the reference rate. Curves are fitted with a discontinuity at October 2015. Circles indicate the normalized rate (rate of enrollees with at least 1 diagnosis in a diagnostic category divided by the September 2015 rate); orange lines, predicted rate using a piecewise linear model; blue lines, predicted rate using locally estimated scatterplot smoothing algorithm; AHRQ-CCS, Agency for Healthcare Research and Quality Clinical Classification System; HHS-HCC, Health and Human Services Hierarchical Condition Category; and *ICD-10-CM*, *International Statistical Classification of Diseases, Tenth Revision, Clinical Modification*.

Figure 3 shows monthly diagnostic category prevalence rates for 6 selected pregnancy-related categories. Additional pregnancy-related categories are included in eFigure 3 in the Supplement. We found no change overall in the broad WHO chapter including complications of pregnancy, childbirth, and the puerperium (eFigure 3 in the Supplement), nor for pregnancies without complications in the HHS-HCC or AHRQ-CCS systems. The AHRQ-CCS system had a large negative level change in other complications of birth (−58.8% [95% CI, −62.6% to −55.0%]) and an increase in other complications of pregnancy (66.6% [95% CI, 61.3% to 71.9%]). No other pregnancy categories examined in the AHRQ-CCS system had large changes in level or trend (eFigure 3 in the Supplement).

Our clinical review noted that the number of obstetrical codes doubled in *ICD-10-CM* vs *ICD-9-CM* and that the *ICD-10-CM* coding system was restructured to remove designation of antepartum, delivery, and postpartum status and to add separate codes to indicate weeks of gestation for ongoing pregnancies or pregnancy results for individuals who are no longer pregnant. Among HHS-HCCs, some conditions, such as breech presentation and previous cesarean delivery, were removed from the category for completed pregnancies with complications, contributing to declines in the prevalence of these diagnostic categories. In contrast, the AHRQ-CCS system adapted to the increased detail and restructuring of the obstetrical codes by adding many more specific codes to its existing categories, resulting in abrupt increases in their prevalence at the *ICD-10-CM* transition.

Findings from sensitivity analyses were broadly consistent with the main analyses. These included using monthly counts of diagnoses in each category as the primary outcome, not using the HHS filtering logic, and substituting the 2015 HHS-HCC software for the 2017 version to set the diagnostic category mappings.

## Discussion

Our interrupted time series analysis and cross-sectional study of commercial claims from 2010 to 2017 revealed striking changes in levels and trends for many diagnostic categories associated with the transition to *ICD-10-CM*. Only small changes in levels or trends were found for the WHO disease chapters, while 16% of HHS-HHCs and AHRQ-CCS categories displayed large and significant level changes, while 9% to 10% displayed large changes in trend.

Our clinical review found that abrupt level changes were largely due to different decisions about which codes to include or exclude in post–*ICD-10-CM* diagnostic categories, such as diabetes coded jointly with hypoglycemia or hyperglycemia leading to a complicated diabetes category, non-STEMI being included (or not) in the AMI category, and breech presentation leading to classification (or not) as a complication of pregnancy. We also identified some changes in coding instructions and coding practice that may have affected disease prevalence trend rates. Many *ICD-10-CM* codes have no corresponding *ICD-9-CM* code. For certain conditions, the new, more specific *ICD-10-CM* codes were rarely used in 2015 and 2016 but began to appear more regularly in 2017. For example, the strong upward trend in diabetes with acute and chronic complications is partially due to increasing use of codes for drug or chemical induced diabetes not available in *ICD-9-CM*. Likewise, refined codes, such as STEMI myocardial infarction of anterior wall (available starting in 2016), only began to appear in 2017, and many new codes for preeclampsia introduced in 2015 were infrequently used before 2017.

Our findings have important implications for interpreting differences in billing-code-derived disease prevalence over time. For epidemiology, it is critical to distinguish between changes in actual disease prevalence and changes in coding behaviors and mappings, which are often responsible for observed changes in coded prevalence. We have seen that 3 separate, widely used classification systems made different distinctions that affected apparent diagnostic category prevalence. Given that HHS payment formulas rely on the prevalence of HCC categories to set billions of dollars in payments, our finding of many large (artifactual) changes in diagnostic category prevalence has potentially large implications for reimbursement.

Although HHS-HCCs are used for the ACA’s marketplace risk adjustment payments, this study did not examine financial outcomes. Because the ACA uses risk adjustment to reallocate the available funds among plans in a given region rather than to decide on the size of the funds available (as is done in Medicare Advantage and Part D), changes in diagnostic category prevalence may have had less financial effect in the marketplace than in Medicare Advantage. Classifications used for bonus or performance evaluation are also vulnerable to large changes in level or trend when preexisting *ICD-9-CM*–derived formulas are used across the *ICD-10-CM* transition. In addition, claims-based analyses of disease prevalence may also be misleading if changes in the underlying classification system are not recognized.

Our study used the earliest mappings accommodating all valid 2017 diagnoses for the included classification systems. In 2019, the WHO, HHS, and AHRQ released new classification systems with expanded diagnostic categories designed for 2019 diagnoses.^17,21,22^ Future research will need to examine changes under these new systems. In the meantime, it is important to understand the current mappings that will continue to be used to set payments and evaluate performance for several more years.

### Limitations

This study has several limitations. First, we only examined the association of diagnostic category prevalence with the *ICD-10-CM* transition for individuals younger than 65 years who were privately insured by employer-sponsored insurance, a population mostly enrolled in relatively generous health plans. Coding patterns and discontinuities in frequencies of diagnostic categories may not generalize to individuals covered by other insurers (eg, the ACA marketplace, Medicare, and Medicaid). Many health care practitioners have been encouraged by their institutions to maximize the capture of patient complexity in their billing. However, our study data came from commercial plans whose payments are rarely risk adjusted for disease prevalence. Specifically, their payments do not generally rely on HHS-HCCs, reducing the explicit incentive to increase the prevalence of well-reimbursed HHS-HCC categories. Second, we did not examine changes at the *ICD-10-CM* transition on coding for particular conditions for specific patients or in category prevalence based on other sources of diagnoses, such as electronic medical records. Third, we used a piecewise linear model to look for changes in level and trend, but for some categories in which diagnostic category prevalence trends are not linear, our model may find changes at the transition when a nonlinear model would not.

## Conclusions

The findings of this interrupted time series analysis and cross-sectional study suggest that the transition to *ICD-10-CM* in October 2015 was associated with changes in levels and trends for most diagnostic categories in 3 common diagnostic classification systems. While the broad categories used in WHO disease chapters showed only small changes at the transition, numerous large changes (ie, ≥20%) in diagnostic category prevalence occurred in the more detailed HHS-HCCs and AHRQ-CCS categories. These 2 classification systems have been widely adopted by health care organizations for many purposes. Given the frequent, large discontinuities in diagnostic category prevalence rates that we identified, predictive models and diagnostic category mappings developed for *ICD-9-CM* should be refined for *ICD-10-CM* data to avoid unintended consequences for health care payment, performance assessment, or disease surveillance.

## Notes

Supplement. **eAppendix.** Statistical Overview **eFigure 1.** Monthly Plots of Selected Population Characteristics **eFigure 2.** Cardiac Chapter for World Health Organization **eFigure 3.** Selected Other Diagnostic Categories for Pregnancy Conditions in World Health Organization, Health and Human Services Hierarchical Condition Categories, and Agency for Healthcare Research and Quality Clinical Classification System With Only Small Changes Associated With October 2015 **eTable 1.** Piecewise Linear Regression Coefficients Predicting 2010 to 2017 Monthly Prevalence for 19 WHO Chapters **eTable 2.** Piecewise Linear Regression Coefficients Predicting 2010 to 2017 Monthly Prevalence for 127 Health and Human Services Hierarchical Condition Categories **eTable 3.** Piecewise Linear Regression Coefficients Predicting 2010 to 2017 Monthly Prevalence for 282 Agency for Healthcare Research and Quality Clinical Classification System Categories
