# Supplementary material for: Diagnostic Category Prevalence in 3 Classification Systems Across the Transition to the International Classification of Diseases, Tenth Revision, Clinical Modification
Source: JAMA Netw Open. 2020 Apr 8;3(4):e202280. doi: 10.1001/jamanetworkopen.2020.2280 (PMC7142382; doi:10.1001/jamanetworkopen.2020.2280)
Supplement: Supplement. — eAppendix. Statistical Overview eFigure 1. Monthly Plots of Selected Population Characteristics eFigure 2. Cardiac Chapter for World Health Organization eFigure 3. Selected Other Diagnostic Categories for Pregnancy Conditions in World Health Organization, Health and Human Services Hierarchical Condition Categories, and Agency for Healthcare Research and Quality Clinical Classification System With Only Small Changes Associated With October 2015 eTable 1. Piecewise Linear Regression Coefficients Predicting 2010 to 2017 Monthly Prevalence for 19 WHO Chapters eTable 2. Piecewise Linear Regression Coefficients Predicting 2010 to 2017 Monthly Prevalence for 127 Health and Human Services Hierarchical Condition Categories eTable 3. Piecewise Linear Regression Coefficients Predicting 2010 to 2017 Monthly Prevalence for 282 Agency for Healthcare Research and Quality Clinical Classification System Categories [file jamanetwopen-3-e202280-s001.pdf]

Supplementary Online Content

Ellis RP, Hsu HE, Song C, et al. Diagnostic category prevalence in 3 classification systems across the transition to the *International Classification of Diseases, Tenth Revision, Clinical Modification*. *JAMA Netw Open.* 2020;3(4):e202280. doi:10.1001/jamanetworkopen.2020.2280

eAppendix. Statistical Overview
eFigure 1. Monthly Plots of Selected Population Characteristics
eFigure 2. Cardiac Chapter for World Health Organization
eFigure 3. Selected Other Diagnostic Categories for Pregnancy Conditions in World Health Organization, Health and Human Services Hierarchical Condition Categories, and Agency for Healthcare Research and Quality Clinical Classification System With Only Small Changes Associated With October 2015
eTable 1. Piecewise Linear Regression Coefficients Predicting 2010 to 2017 Monthly Prevalence for 19 WHO Chapters
eTable 2. Piecewise Linear Regression Coefficients Predicting 2010 to 2017 Monthly Prevalence for 127 Health and Human Services Hierarchical Condition Categories
eTable 3. Piecewise Linear Regression Coefficients Predicting 2010 to 2017 Monthly Prevalence for 282 Agency for Healthcare Research and Quality Clinical Classification System Categories

This supplementary material has been provided by the authors to give readers additional information about their work.

© 2020 Ellis RP et al. *JAMA Network Open.*

eAppendix. Statistical Overview

This appendix contains details about the piecewise linear regression specification. Three additional appendices present full results for all three classification systems.

Outcome variable: We started by counting the number of eligible individuals with at least one diagnosis in a classification category in each month and dividing this sum by the number of people eligible in that month. This monthly category prevalence rate was expressed as a rate per 10,000. Because months vary in their number of days, each raw monthly rate was standardized by dividing by the number of days in the month and multiplying by 30.437, the average number of days per month over the study period. Regressions were conducted using outcomes in levels, before normalization.

Model Specification

We estimated piecewise linear models of the form:

$$Outcome = \alpha + \beta t + \gamma D_{ICD10} + \delta D_{ICD10}(t - 70) + \sum_{m=2}^{12} \mu_m * D_m + \varepsilon_t$$

Where:

t = time in months from January 2010, ranging from 1 to 96,

D_{ICD10} = 1 if t ≥ 70, else 0, (since October 2015 = month 70),

m = index 1 – 12 of calendar months,

D_m = monthly dummy variables to pick up monthly seasonality,

α, β, γ, δ, μ_m are parameters to be estimated, and

ε_t is a monthly error term.

In order to facilitate the interpretation of results as percentage changes, we normalized all regression coefficients and their standard errors by dividing them by the adjusted average rate in September 2015, the final month before the introduction of ICD-10-CM. This enabled us to interpret all model parameters in terms of percentage changes from September 2015. The term α is the predicted pre-sample (month 0) rate (as a percent of the September 2015 rate); β is the incremental percent change for each month before ICD-10-CM; and γ is the percent change that occurred between and September to October 2015, which we call the "level change in October 2015". Because we started

© 2020 Ellis RP et al. *JAMA Network Open.*

the time trend at month 70 and divided by 26, the number of months observed post ICD-10, the δ coefficient captures the percentage “time trend effect” that occurred during the 26 months following ICD-10-CM. Not estimated directly by the specification but easily calculated is $\gamma + \delta * 26$ which is the “cumulative effect of the level and trend change” from ICD10 to the end of our sample. These parameters and their standard errors are presented in each appendix table for all diagnostic categories.

Standard errors from the regression (and the linear combination showing the cumulative effect) were used to calculate p-values and confidence intervals for each parameter. Because we conducted separate tests for each of the several disease categories in each classification system, we used the Bonferroni correction to avoid identifying too many changes that could be due to chance alone. Given the N= 19, 127 and 282 categories for WHO, HHS-HCC and AHRQ-CCS, respectively the p-values that we interpreted as significant at the 5% level are .0027, .0004 and .00017 for the three classification systems; analogously, we used the following two sided t-statistic multiples of the standard error to calculate confidence intervals: -3.104, -3.698, and -3.931 for WHO, HHS, and CCS systems, respectively.

© 2020 Ellis RP et al. *JAMA Network Open.*

eFigure 1. Monthly Plots of Selected Population Characteristics

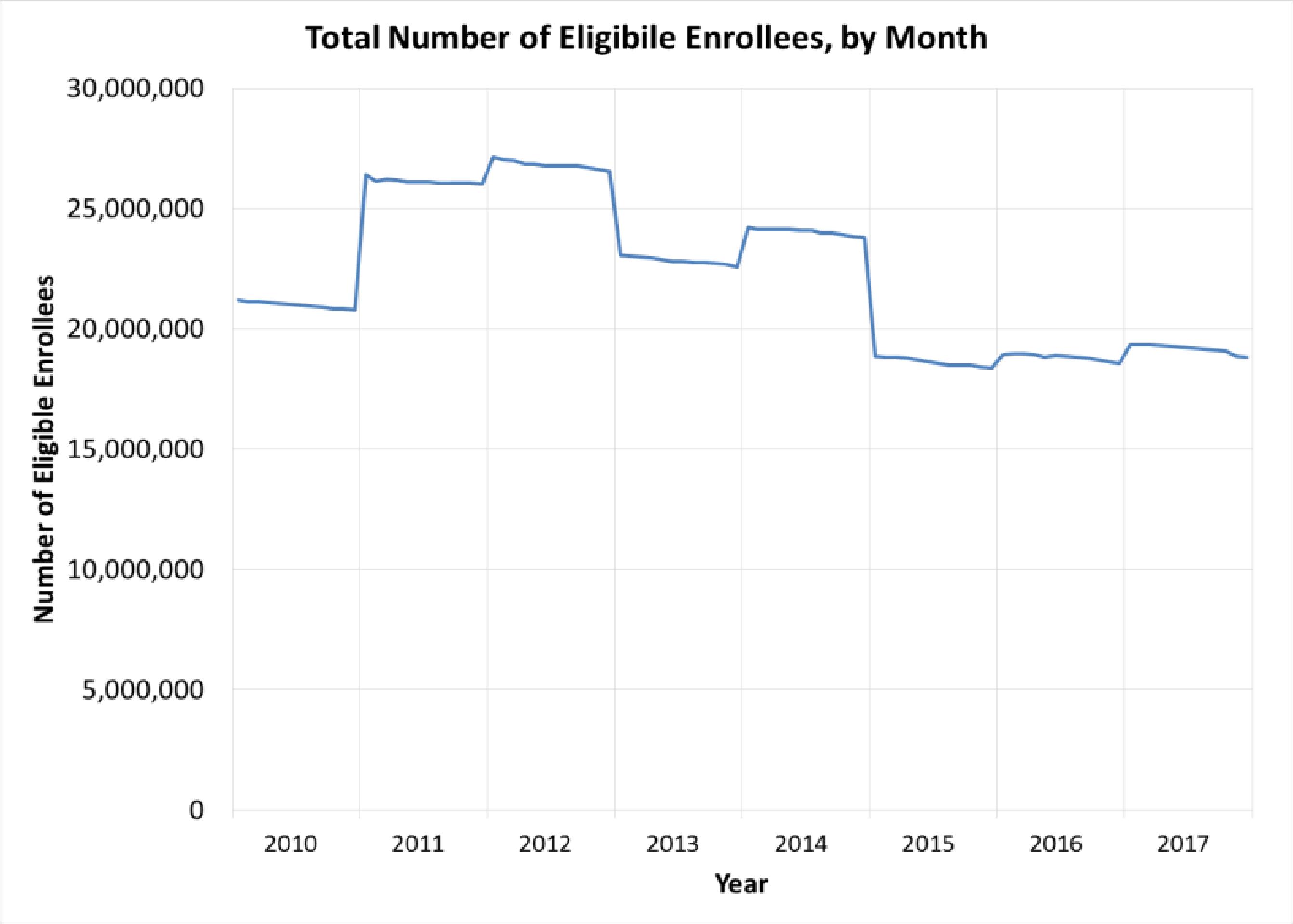

© 2020 Ellis RP et al. *JAMA Network Open.*

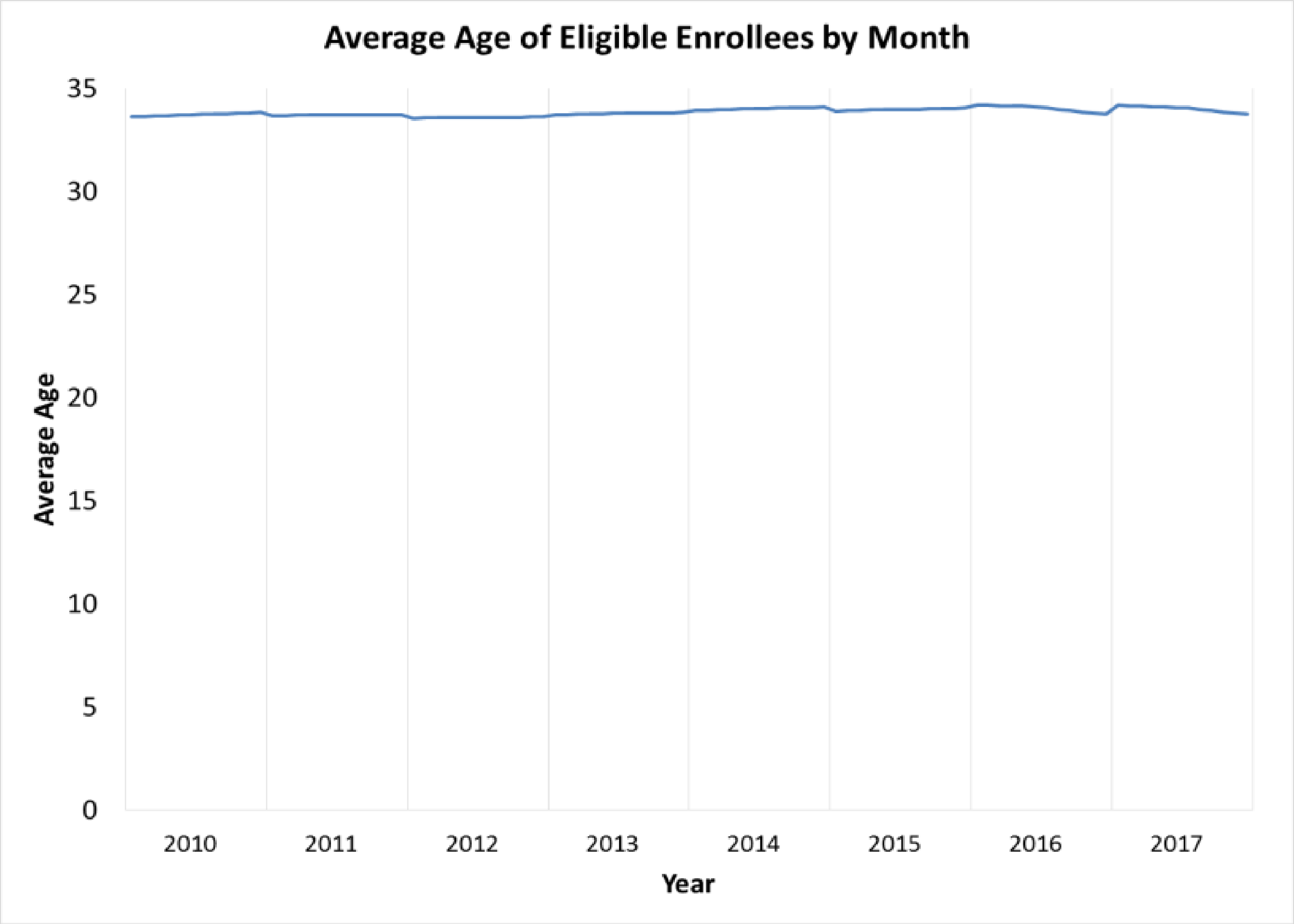

© 2020 Ellis RP et al. *JAMA Network Open.*

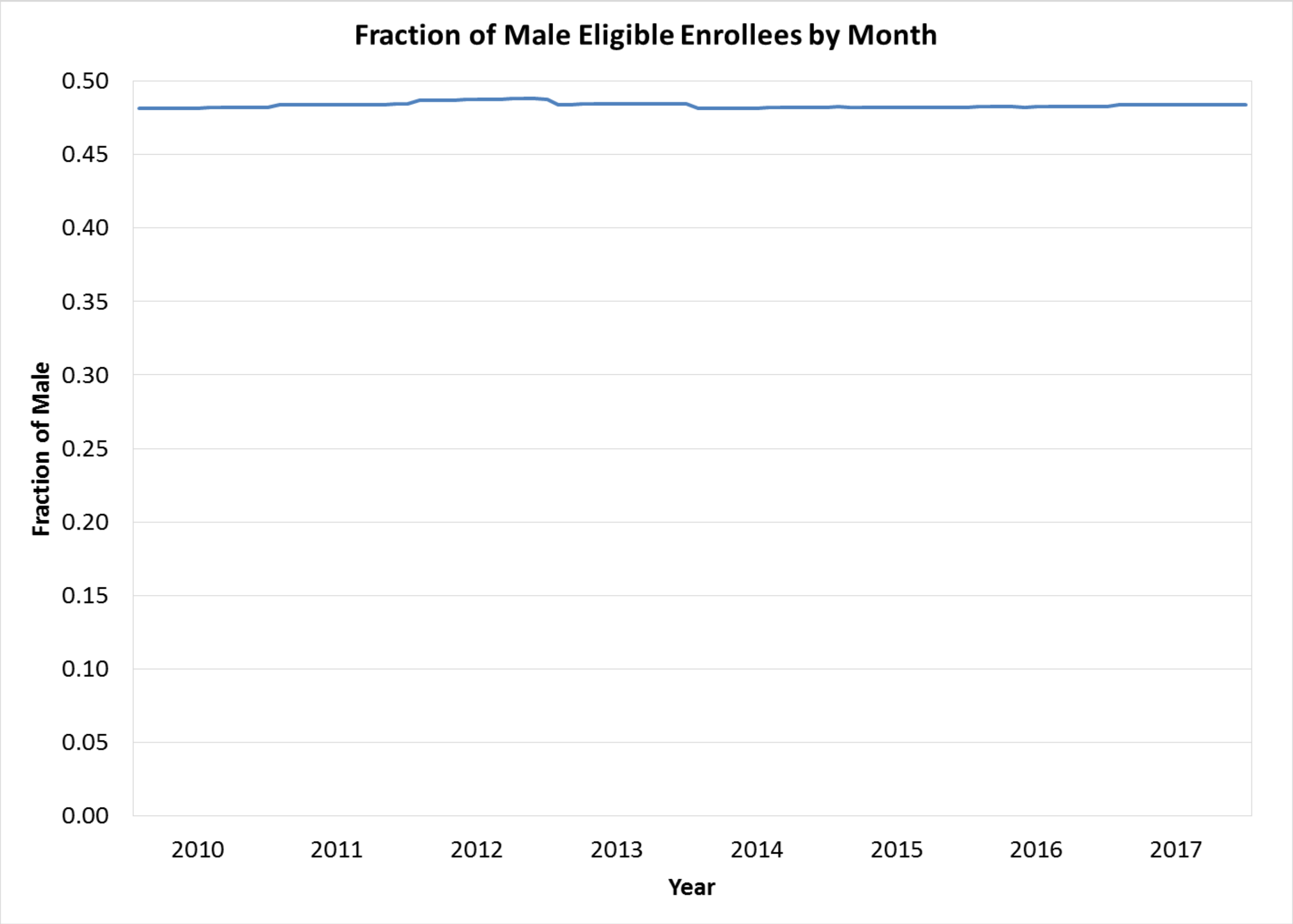

© 2020 Ellis RP et al. *JAMA Network Open.*

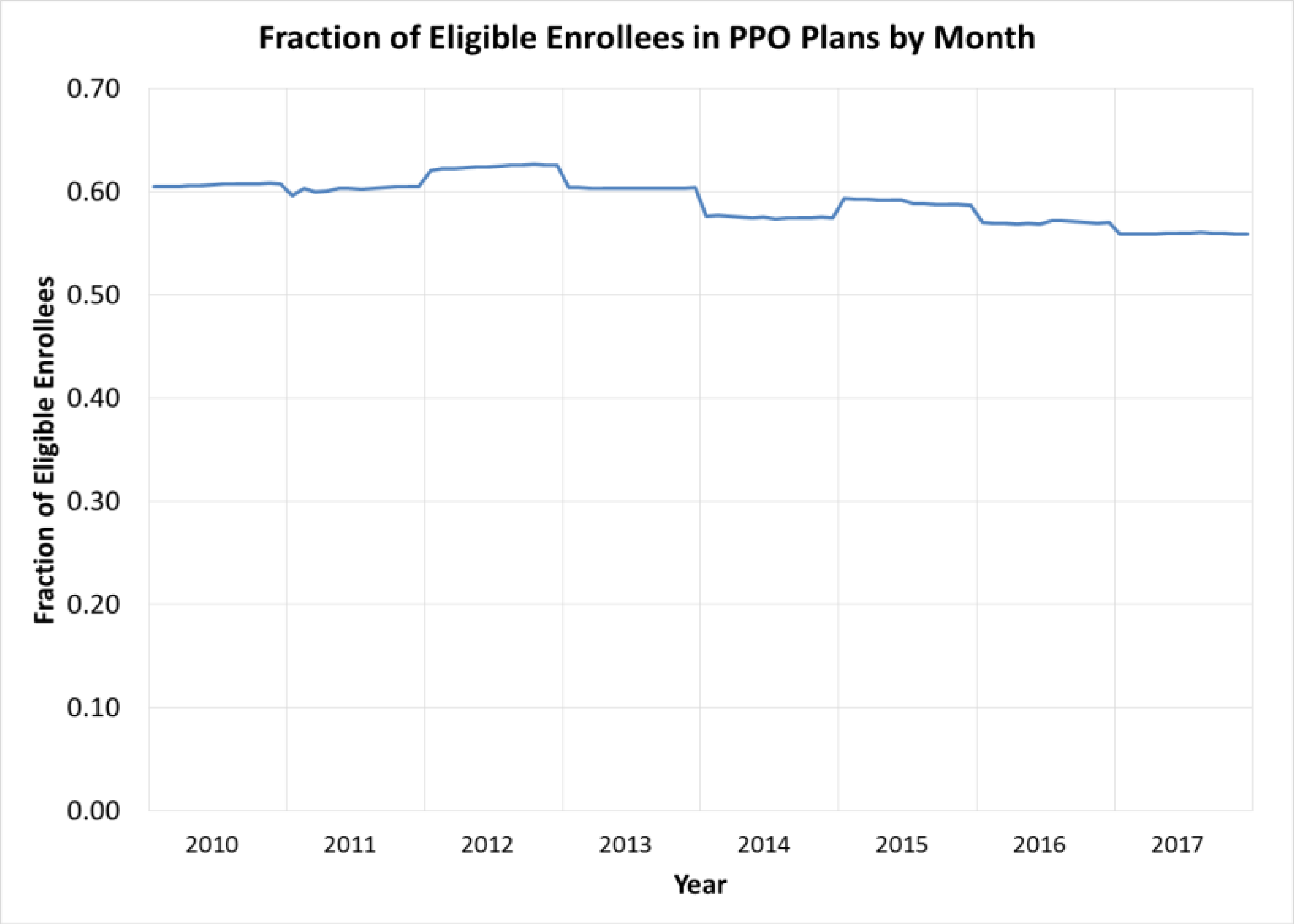

© 2020 Ellis RP et al. *JAMA Network Open.*

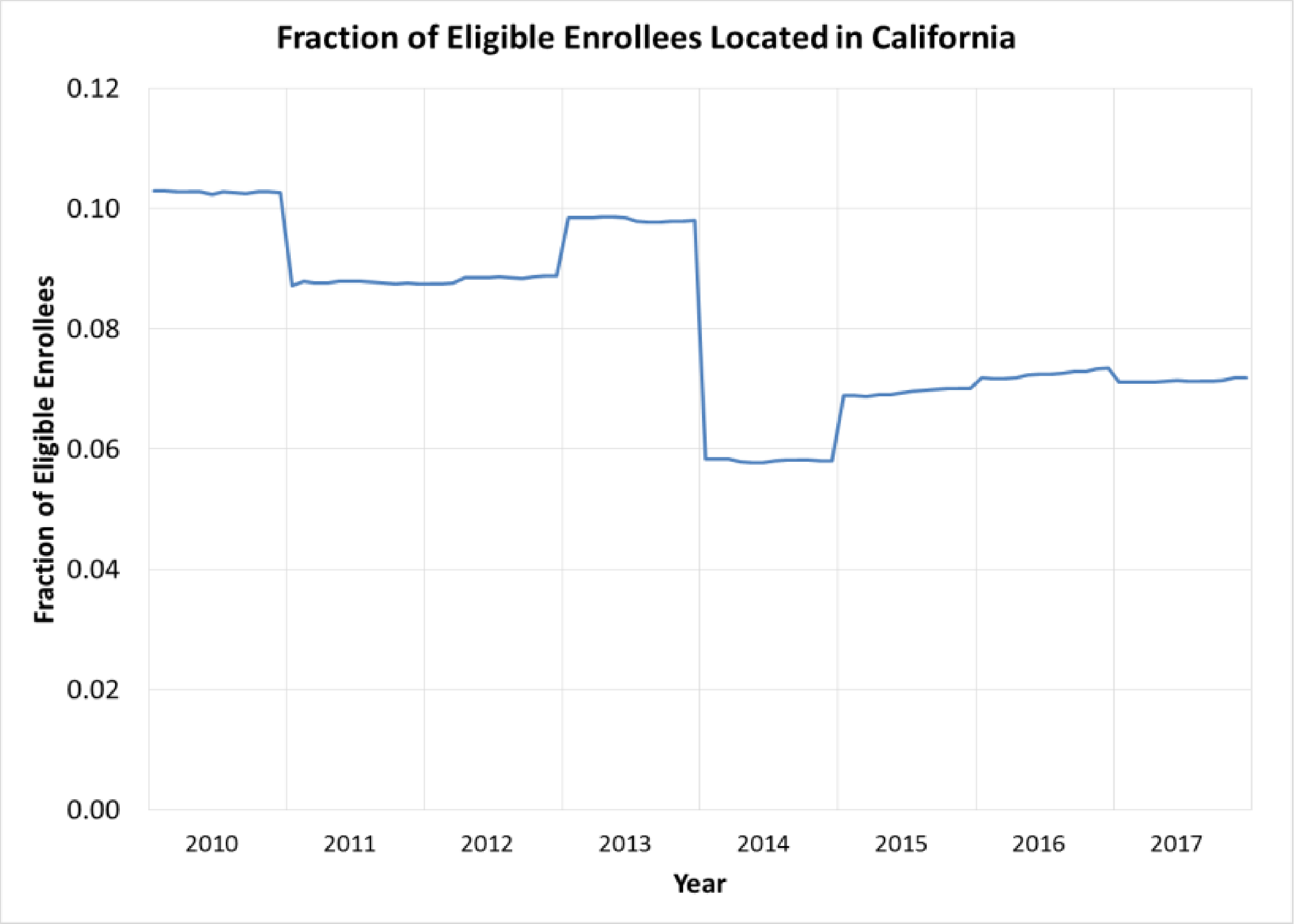

© 2020 Ellis RP et al. *JAMA Network Open.*

eFigure 2 Cardiac Chapter for World Health Organization

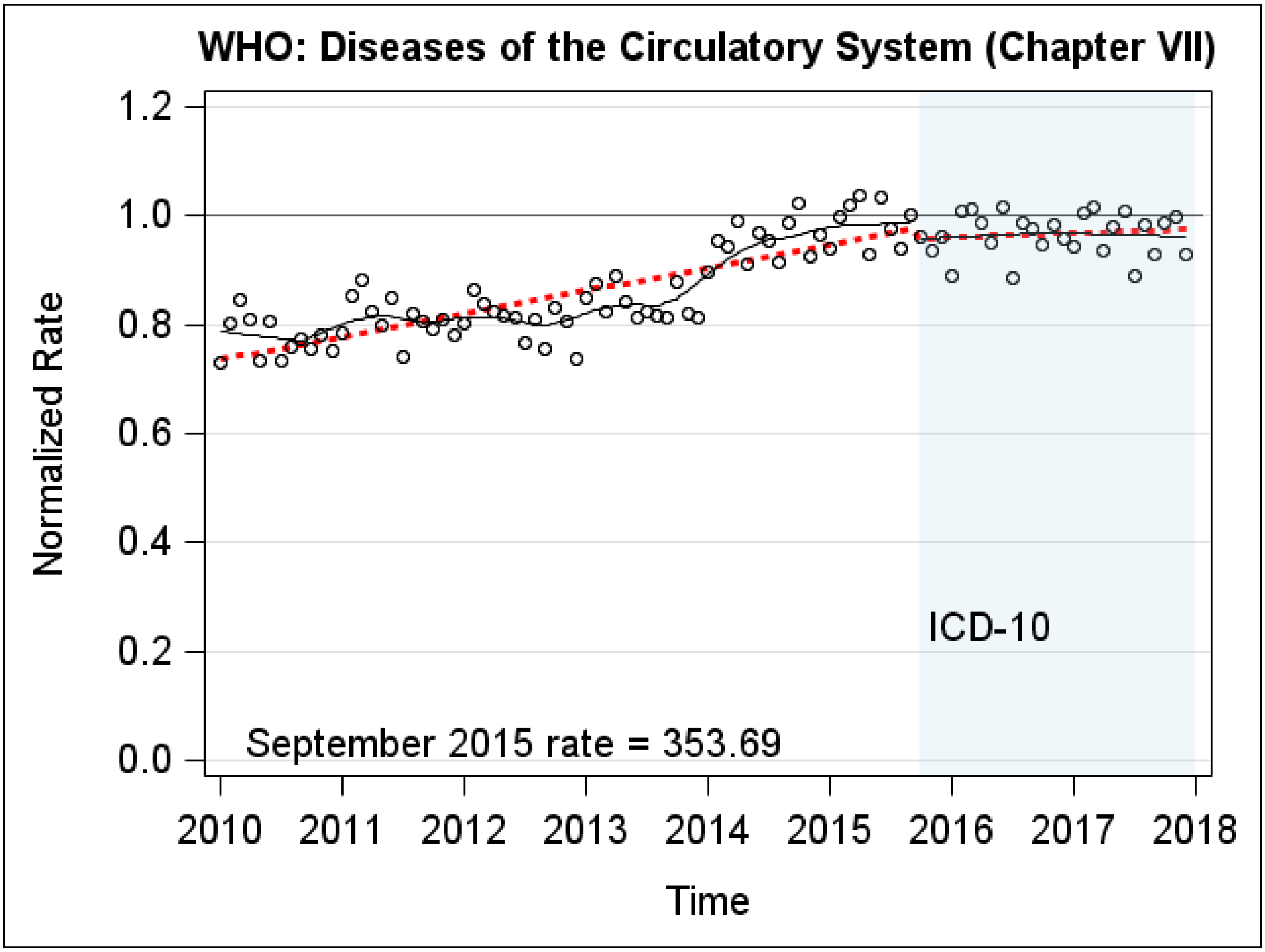

Notes: The diagnostic category prevalence rate per 10,000 enrollees in September 2015 is shown in the lower left corner of each panel. The "normalized rate" (plotted as o) is the rate of enrollees with at least one diagnosis in a diagnostic category divided by the September 2015 rate. Both ···· and — are curves fitted with a discontinuity at October 2015: ···· using a piecewise linear model and –– using a LOESS smoothing algorithm.

© 2020 Ellis RP et al. *JAMA Network Open.*

eFigure 3 Selected Other Diagnostic Categories for Pregnancy Conditions in World Health Organization, Health and Human Services Hierarchical Condition Categories, and Agency for Healthcare Research and Quality Clinical Classification System With Only Small Changes Associated With October 2015

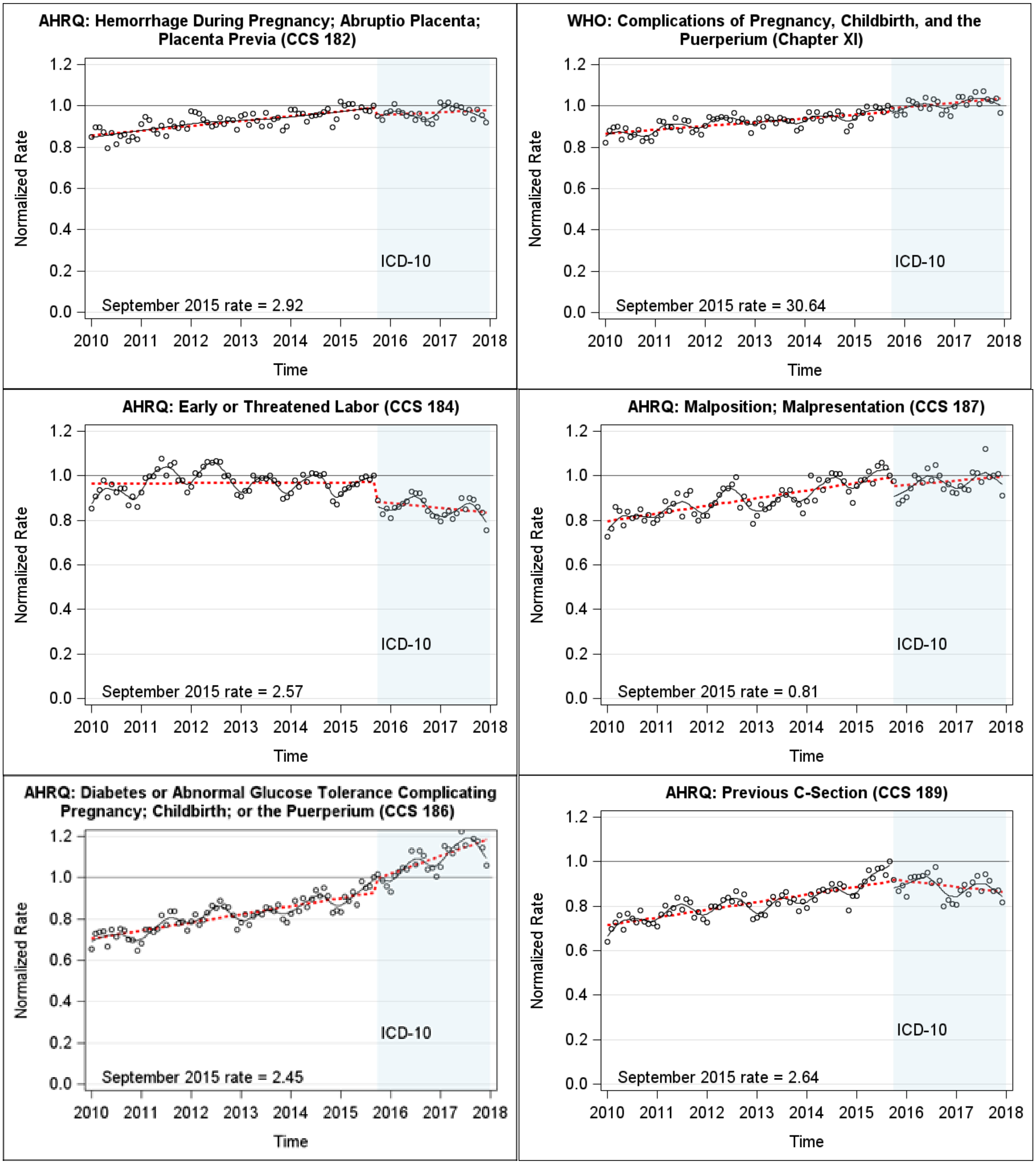

Notes: The diagnostic category prevalence rate per 10,000 enrollees in September 2015 is shown in the lower left corner of each panel. The "normalized rate" (plotted as o) is the rate of enrollees with at least one diagnosis in a diagnostic category divided by the September 2015 rate. Both ···· and — are curves fitted with a discontinuity at October 2015: ···· using a piecewise linear model and –– using a LOESS smoothing algorithm.

© 2020 Ellis RP et al. *JAMA Network Open.*

eTable 1. Piecewise Linear Regression Coefficients Predicting 2010 to 2017 Monthly Prevalence for 19 WHO Chapters

Gray-shaded regions are statistically significant; darker (blue) shaded areas are statistically significant and large, that is, |coefficient| ≥ 0.2.
95% Confidence intervals (CI) and statistical significance calculated using the Bonferroni correction.

		Sept 2015 prevalence per 10,000 enrollees	Intercept: Estimated relative rate in Dec 2009 (CI)	Slope: Estimated trend rate before ICD-10 (CI)	Level change in October 2015 (CI)	Time trend change Oct 2015 – Dec 2017 (CI)	Cumulative effect of level and time trend changes (CI)	F-test: For a straight line trend (p-value)
**WHO Chapters**		**9/15 Rate**	α	β	γ	δ	$\gamma+\delta$	F: γ=δ=0
1	Infectious and Parasitic Diseases	152.97 (152.33, 153.61)	0.88 (0.82, 0.93)	0.003 (0.002, 0.003)	-0.123 (-0.187, -0.06)	0.043 (-0.05, 0.136)	-0.081 (-0.156, -0.005)	0.00000
2	Neoplasms	172.79 (172.11, 173.47)	0.7 (0.65, 0.74)	0.003 (0.002, 0.003)	-0.007 (-0.055, 0.041)	-0.014 (-0.085, 0.058)	-0.021 (-0.078, 0.037)	0.56067
3	Endocrine, Nutritional and Metabolic Diseases, and Immunity Disorders	458.18 (457.09, 459.27)	0.6 (0.55, 0.64)	0.005 (0.004, 0.006)	0.007 (-0.041, 0.055)	-0.056 (-0.127, 0.015)	-0.049 (-0.106, 0.009)	0.04678
4	Diseases of the Blood and Blood-Forming Organs	55.86 (55.47, 56.25)	0.58 (0.53, 0.62)	0.005 (0.004, 0.006)	0.098 (0.049, 0.148)	-0.014 (-0.088, 0.059)	0.084 (0.025, 0.143)	0.00000
5	Mental Disorders	453.21 (452.13, 454.29)	0.69 (0.66, 0.72)	0.004 (0.004, 0.0045)	-0.069 (-0.098, -0.039)	0.048 (0.004, 0.091)	-0.021 (-0.056, 0.014)	0.00000
6	Diseases of the Nervous System and Sense Organs	413.78 (412.74, 414.82)	0.83 (0.79, 0.87)	0.003 (0.002, 0.003)	0.149 (0.101, 0.196)	0.001 (-0.069, 0.071)	0.15 (0.093, 0.206)	0.00000
7	Diseases of the Circulatory System	353.69 (352.73, 354.65)	0.71 (0.67, 0.76)	0.004 (0.003, 0.004)	-0.019 (-0.07, 0.031)	-0.075 (-0.149, -0.001)	-0.094 (-0.154, -0.034)	0.00010
8	Diseases of the Respiratory System	490.42 (489.29, 491.55)	1.22 (1.14, 1.29)	0.001 (0.0001, 0.002)	0.015 (-0.068, 0.097)	-0.005 (-0.127, 0.117)	0.01 (-0.089, 0.108)	0.85749
9	Diseases of the Digestive System	201.36 (200.63, 202.09)	0.71 (0.67, 0.76)	0.004 (0.003, 0.004)	0.056 (0.008, 0.104)	-0.048 (-0.119, 0.023)	0.008 (-0.049, 0.065)	0.00506
10	Diseases of the Genitourinary System	250.07 (249.26, 250.88)	0.77 (0.73, 0.8)	0.002 (0.002, 0.003)	-0.011 (-0.054, 0.032)	-0.065 (-0.128, -0.001)	-0.076 (-0.127, -0.025)	0.00030
11	Complications of Pregnancy, Childbirth, and the Puerperium	30.64 (30.35, 30.93)	0.85 (0.82, 0.87)	0.002 (0.001, 0.002)	0.016 (-0.01, 0.043)	0.008 (-0.032, 0.047)	0.024 (-0.008, 0.056)	0.03350
12	Diseases of the Skin and Subcutaneous Tissue	279.92 (279.06, 280.78)	0.71 (0.67, 0.76)	0.003 (0.002, 0.003)	0.029 (-0.016, 0.074)	-0.015 (-0.081, 0.052)	0.014 (-0.039, 0.068)	0.15810
13	Diseases of the Musculoskeletal System and Connective Tissue	691.57 (690.25, 692.89)	0.77 (0.74, 0.8)	0.002 (0.002, 0.003)	0.013 (-0.02, 0.046)	-0.035 (-0.084, 0.013)	-0.022 (-0.062, 0.017)	0.11976
14	Congenital Anomalies	31.64 (31.35, 31.93)	0.6 (0.55, 0.64)	0.004 (0.004, 0.005)	-0.058 (-0.107, -0.009)	-0.034 (-0.107, 0.038)	-0.093 (-0.151, -0.034)	0.00000
15	Certain Conditions Originating In the Perinatal Period	8.63 (8.48, 8.78)	0.78 (0.74, 0.82)	0.002 (0.001, 0.002)	0.024 (-0.019, 0.066)	0.091 (0.028, 0.154)	0.115 (0.064, 0.165)	0.00000
16	Symptoms, Signs, and Ill-Defined Conditions	653.1 (651.81, 654.39)	0.72 (0.67, 0.77)	0.005 (0.004, 0.005)	0.044 (-0.008, 0.096)	-0.039 (-0.116, 0.038)	0.005 (-0.057, 0.067)	0.06020
17	Injury and Poisoning	257.9 (257.07, 258.73)	0.81 (0.78, 0.84)	0.001 (0.0001, 0.0009)	-0.096 (-0.128, -0.064)	-0.012 (-0.06, 0.035)	-0.108 (-0.147, -0.07)	0.00000
18	Factors Influencing Health Status and Contact with Health Services	903.86 (902.37, 905.35)	0.57 (0.54, 0.61)	0.004 (0.004, 0.005)	0.040 (-0.003, 0.083)	0.032 (-0.032, 0.096)	0.072 (0.02, 0.124)	0.00009

© 2020 Ellis RP et al. *JAMA Network Open.*

19	External Causes of Injury and Poisoning	36.69 (36.37, 37.01)	0.3 (0.23, 0.38)	0.006 (0.005, 0.007)	0.121 (0.039, 0.202)	-0.167 (-0.287, -0.047)	-0.046 (-0.143, 0.051)	0.00008
**WHO Chapters**		**9/15 Rate**	$\boldsymbol{\alpha}$	$\boldsymbol{\beta}$	$\boldsymbol{\gamma}$	$\boldsymbol{\delta}$	$\boldsymbol{\gamma + \delta}$	$\boldsymbol{F}$: $\boldsymbol{\gamma}$=$\boldsymbol{\delta}$=0
ALL	# of chapters (out of 19), where coefficients are statistically significant (that is, P < 0.05/19)		19	19	8	5	9	11
ALL	# of chapters with coefficients that are statistically significant and large (|coef| ≥20%)		0	0	0	0	0	0

© 2020 Ellis RP et al. *JAMA Network Open.*

eTable 2. Piecewise Linear Regression Coefficients Predicting 2010 to 2017 Monthly Prevalence for 127 Health and Human Services Hierarchical Condition Categories

Gray-shaded regions are statistically significant; darker (blue) shaded areas are statistically significant and large, that is, |coefficient| ≥ 0.2.
Confidence intervals (CI) and statistical significance calculated using the Bonferroni correction, corresponding to P<(.05/127)=0.0004

		Sept 2015 prevalence per 10,000 enrollees	Intercept: Estimated relative rate in Dec 2009 (CI)	Slope: Estimated trend rate before ICD-10 (CI)	Level change in October 2015 (CI)	Time trend change Oct 2015 – Dec 2017 (CI)	Cumulative effect of level and time trend changes (CI)	F-test: For a straight line trend (p-value)
**HCC**	**HCC Label**	**9/15 Rate**	α	β	γ	δ	$\gamma + \delta$	F: γ=δ=0
1	HIV/AIDS	3.75 (3.65, 3.85)	0.81 (0.75, 0.87)	0.002 (0.0012, 0.0029)	0.0337 (-0.032, 0.099)	0.105 (0.01, 0.201)	0.139 (0.061, 0.217)	0.00000
2	Septicemia, Sepsis, Systemic Inflammatory Response Syndrome/Shock	2.63 (2.55, 2.71)	0.47 (0.42, 0.52)	0.0063 (0.0055, 0.007)	0.0784 (0.021, 0.136)	-0.104 (-0.189, -0.02)	-0.026 (-0.094, 0.043)	0.00001
3	Central Nervous System Infections, Except Viral Meningitis	0.64 (0.6, 0.68)	0.59 (0.53, 0.65)	0.0036 (0.0027, 0.0046)	-0.0174 (-0.086, 0.052)	-0.076 (-0.177, 0.026)	-0.093 (-0.175, -0.011)	0.00082
4	Viral or Unspecified Meningitis	0.42 (0.39, 0.45)	0.57 (0.49, 0.65)	0.0006 (-0.0006, 0.0017)	-0.0245 (-0.113, 0.064)	-0.069 (-0.198, 0.061)	-0.093 (-0.198, 0.012)	0.00970
6	Opportunistic Infections	0.67 (0.63, 0.71)	0.49 (0.43, 0.56)	0.0058 (0.0048, 0.0067)	0.1466 (0.077, 0.216)	-0.027 (-0.129, 0.076)	0.12 (0.037, 0.203)	0.00000
8	Metastatic Cancer	6.72 (6.58, 6.86)	0.56 (0.52, 0.6)	0.0059 (0.0053, 0.0065)	0.0365 (-0.007, 0.08)	0.04 (-0.024, 0.105)	0.077 (0.025, 0.129)	0.00000
9	Lung, Brain, and Other Severe Cancers, Including Pediatric Acute Lymphoid Leukemia	7.37 (7.23, 7.51)	0.85 (0.83, 0.88)	0.0014 (0.001, 0.0018)	-0.0033 (-0.034, 0.027)	0.002 (-0.043, 0.047)	-0.001 (-0.038, 0.035)	0.92578
10	Non-Hodgkin`s Lymphomas and Other Cancers and Tumors	4.78 (4.67, 4.89)	0.91 (0.87, 0.94)	0.001 (0.0004, 0.0015)	-0.0056 (-0.047, 0.036)	0.033 (-0.028, 0.095)	0.028 (-0.022, 0.077)	0.13145
11	Colorectal, Breast (Age < 50), Kidney, and Other Cancers	11.98 (11.8, 12.16)	0.89 (0.86, 0.93)	0.0006 (0.0001, 0.0011)	-0.0094 (-0.048, 0.029)	0.027 (-0.031, 0.084)	0.017 (-0.029, 0.063)	0.27619
12	Breast (Age 50+) and Prostate Cancer, Benign/ Uncertain Brain Tumors; Other Cancers and Tumors	23 (22.75, 23.25)	0.82 (0.78, 0.87)	0.0017 (0.0011, 0.0023)	-0.0102 (-0.057, 0.036)	-0.012 (-0.08, 0.057)	-0.022 (-0.077, 0.034)	0.33494
13	Thyroid Cancer, Melanoma, Neurofibromatosis, and Other Cancers and Tumors	6.23 (6.1, 6.36)	0.67 (0.62, 0.72)	0.0041 (0.0034, 0.0048)	-0.0265 (-0.078, 0.025)	-0.103 (-0.179, -0.027)	-0.13 (-0.191, -0.068)	0.00000
18	Pancreas Transplant Status/Complications	0.09 (0.07, 0.11)	0.51 (0.42, 0.61)	0.005 (0.0036, 0.0064)	-0.0459 (-0.152, 0.06)	-0.178 (-0.334, -0.021)	-0.224 (-0.35, -0.097)	0.00000
19	Diabetes with Acute Complications	0.89 (0.84, 0.94)	0.65 (0.57, 0.73)	0.0038 (0.0027, 0.005)	0.028 (-0.058, 0.114)	0.244 (0.117, 0.371)	0.272 (0.169, 0.375)	0.00000
20	Diabetes with Chronic Complications	27.52 (27.25, 27.79)	0.52 (0.45, 0.59)	0.0048 (0.0037, 0.0059)	0.924 (0.842, 1.005)	0.237 (0.117, 0.357)	1.161 (1.064, 1.258)	0.00000
21	Diabetes without Complication	116.77 (116.21, 117.33)	0.71 (0.65, 0.76)	0.0037 (0.0028, 0.0045)	-0.191 (-0.253, -0.129)	-0.076 (-0.167, 0.014)	-0.267 (-0.341, -0.194)	0.00000
23	Protein-Calorie Malnutrition	1.17 (1.11, 1.23)	0.5 (0.44, 0.55)	0.0059 (0.0051, 0.0067)	0.0622 (0.001, 0.123)	0.056 (-0.034, 0.145)	0.118 (0.046, 0.191)	0.00000

© 2020 Ellis RP et al. *JAMA Network Open.*

HCC	HCC Label	9/15 Rate	α	β	γ	δ	$\gamma+\delta$	F: γ=δ=0
26	Mucopolysaccharidosis	0.04 (0.03, 0.05)	0.35 (0.24, 0.46)	0.0088 (0.0072, 0.0105)	0.1143 (-0.007, 0.236)	-0.302 (-0.481, -0.123)	-0.188 (-0.333, -0.043)	0.00000
27	Lipidoses and Glycogenosis	0.27 (0.24, 0.3)	0.77 (0.68, 0.86)	0.004 (0.0026, 0.0053)	-0.4692 (-0.567, -0.372)	-0.036 (-0.179, 0.108)	-0.505 (-0.621, -0.388)	0.00000
28	Congenital Metabolic Disorders, Not Elsewhere Classified	1.41 (1.35, 1.47)	0.33 (0.27, 0.4)	0.0084 (0.0074, 0.0093)	0.0278 (-0.042, 0.098)	-0.048 (-0.151, 0.055)	-0.02 (-0.104, 0.064)	0.24389
29	Amyloidosis, Porphyria, and Other Metabolic Disorders	0.66 (0.62, 0.7)	0.53 (0.47, 0.6)	0.0058 (0.0048, 0.0068)	-0.0035 (-0.08, 0.073)	-0.009 (-0.121, 0.103)	-0.012 (-0.103, 0.079)	0.88944
30	Adrenal, Pituitary, and Other Significant Endocrine Disorders	8.49 (8.34, 8.64)	0.49 (0.44, 0.55)	0.0067 (0.0058, 0.0075)	0.0181 (-0.044, 0.08)	-0.079 (-0.17, 0.012)	-0.061 (-0.135, 0.013)	0.00915
34	Liver Transplant Status/Complications	0.4 (0.37, 0.43)	0.69 (0.62, 0.76)	0.0044 (0.0033, 0.0054)	0.0435 (-0.036, 0.123)	-0.078 (-0.195, 0.039)	-0.034 (-0.129, 0.06)	0.06128
35	End-Stage Liver Disease	1.01 (0.96, 1.06)	0.52 (0.46, 0.58)	0.0062 (0.0054, 0.0071)	-0.0167 (-0.081, 0.048)	-0.066 (-0.161, 0.029)	-0.083 (-0.159, -0.006)	0.00149
36	Cirrhosis of Liver	2.37 (2.29, 2.45)	0.59 (0.54, 0.63)	0.0053 (0.0046, 0.006)	-0.0631 (-0.115, -0.011)	-0.072 (-0.149, 0.004)	-0.135 (-0.197, -0.073)	0.00000
37	Chronic Hepatitis	3.34 (3.24, 3.44)	0.76 (0.68, 0.84)	0.0027 (0.0016, 0.0039)	-0.0073 (-0.096, 0.081)	-0.186 (-0.316, -0.056)	-0.193 (-0.298, -0.088)	0.00000
38	Acute Liver Failure/Disease, Including Neonatal Hepatitis	0.41 (0.38, 0.44)	0.62 (0.56, 0.68)	0.0033 (0.0025, 0.0042)	-0.0739 (-0.139, -0.009)	-0.036 (-0.131, 0.059)	-0.11 (-0.187, -0.033)	0.00000
41	Intestine Transplant Status/Complications	0.01 (0, 0.02)	1.38 (1.06, 1.71)	-0.0033 (-0.0082, 0.0016)	-0.2097 (-0.575, 0.155)	0.249 (-0.288, 0.786)	0.039 (-0.397, 0.475)	0.12596
42	Peritonitis/Gastrointestinal Perforation/Necrotizing Enterocolitis	0.86 (0.81, 0.91)	0.63 (0.59, 0.68)	0.004 (0.0033, 0.0047)	-0.0401 (-0.091, 0.01)	-0.052 (-0.126, 0.023)	-0.092 (-0.152, -0.031)	0.00000
45	Intestinal Obstruction	2.05 (1.98, 2.12)	0.79 (0.71, 0.87)	0.003 (0.0018, 0.0042)	0.1026 (0.014, 0.191)	-0.22 (-0.35, -0.09)	-0.117 (-0.223, -0.012)	0.00000
46	Chronic Pancreatitis	0.66 (0.62, 0.7)	0.7 (0.64, 0.75)	0.0032 (0.0025, 0.004)	0.0365 (-0.022, 0.096)	0.012 (-0.075, 0.099)	0.049 (-0.022, 0.119)	0.01194
47	Acute Pancreatitis/Other Pancreatic Disorders and Intestinal Malabsorption	5.83 (5.7, 5.96)	0.5 (0.43, 0.57)	0.0065 (0.0054, 0.0075)	-0.4274 (-0.506, -0.349)	0.245 (0.129, 0.36)	-0.183 (-0.276, -0.089)	0.00000
48	Inflammatory Bowel Disease	10.98 (10.81, 11.15)	0.68 (0.64, 0.73)	0.0041 (0.0034, 0.0048)	0.0332 (-0.018, 0.084)	0.028 (-0.047, 0.103)	0.061 (0, 0.122)	0.00080
54	Necrotizing Fasciitis	0.07 (0.06, 0.08)	0.65 (0.54, 0.77)	0.0057 (0.0039, 0.0074)	-0.0945 (-0.225, 0.036)	-0.059 (-0.252, 0.134)	-0.153 (-0.31, 0.003)	0.00063
55	Bone/Joint/Muscle Infections/Necrosis	2.42 (2.34, 2.5)	0.67 (0.62, 0.72)	0.0037 (0.003, 0.0044)	0.0136 (-0.039, 0.066)	-0.078 (-0.155, -0.001)	-0.064 (-0.127, -0.002)	0.00127
56	Rheumatoid Arthritis and Specified Autoimmune Disorders	19.09 (18.86, 19.32)	0.72 (0.67, 0.77)	0.0032 (0.0025, 0.0039)	0.0505 (-0.003, 0.104)	-0.063 (-0.142, 0.016)	-0.012 (-0.076, 0.051)	0.00407

© 2020 Ellis RP et al. *JAMA Network Open.*

HCC	HCC Label	9/15 Rate	α	β	γ	δ	$\gamma+\delta$	F: $\gamma=\delta=0$
57	Systemic Lupus Erythematosus and Other Autoimmune Disorders	9.94<br>(9.78, 10.1)	0.64<br>(0.6, 0.69)	0.0044<br>(0.0037, 0.0051)	-0.0089<br>(-0.062, 0.044)	-0.064<br>(-0.143, 0.014)	-0.073<br>(-0.137, -0.01)	0.00065
61	Osteogenesis Imperfecta and Other Osteodystrophies	0.16<br>(0.14, 0.18)	0.5<br>(0.4, 0.59)	0.0055<br>(0.004, 0.0069)	0.1672<br>(0.061, 0.274)	0.015<br>(-0.142, 0.171)	0.182<br>(0.055, 0.309)	0.00000
62	Congenital/Developmental Skeletal and Connective Tissue Disorders	1.54<br>(1.48, 1.6)	0.54<br>(0.48, 0.6)	0.005<br>(0.0041, 0.0059)	0.0339<br>(-0.032, 0.1)	0.076<br>(-0.022, 0.173)	0.11<br>(0.03, 0.189)	0.00002
63	Cleft Lip/Cleft Palate	0.45<br>(0.42, 0.48)	0.76<br>(0.7, 0.82)	0.0037<br>(0.0028, 0.0046)	-0.0187<br>(-0.085, 0.048)	-0.016<br>(-0.114, 0.082)	-0.035<br>(-0.114, 0.045)	0.23603
64	Major Congenital Anomalies of Diaphragm, Abdominal Wall, and Esophagus, Age < 2	0.06<br>(0.05, 0.07)	0.54<br>(0.4, 0.69)	0.0039<br>(0.0017, 0.0061)	-0.0315<br>(-0.194, 0.131)	0.374<br>(0.136, 0.613)	0.343<br>(0.149, 0.536)	0.00000
66	Hemophilia	0.12<br>(0.1, 0.14)	0.75<br>(0.66, 0.84)	0.0029<br>(0.0015, 0.0042)	0.0429<br>(-0.057, 0.143)	-0.037<br>(-0.184, 0.11)	0.006<br>(-0.113, 0.125)	0.33259
67	Myelodysplastic Syndromes and Myelofibrosis	0.36<br>(0.33, 0.39)	0.93<br>(0.88, 0.98)	0.0002<br>(-0.0006, 0.001)	0.0006<br>(-0.058, 0.059)	0.035<br>(-0.051, 0.12)	0.035<br>(-0.034, 0.105)	0.21887
68	Aplastic Anemia	0.17<br>(0.15, 0.19)	1.24<br>(1.12, 1.36)	-0.005<br>(-0.0068, -0.0033)	0.1913<br>(0.061, 0.321)	0.176<br>(-0.015, 0.368)	0.368<br>(0.212, 0.523)	0.00000
69	Acquired Hemolytic Anemia, Including Hemolytic Disease of Newborn	0.43<br>(0.4, 0.46)	0.57<br>(0.51, 0.62)	0.0049<br>(0.0041, 0.0057)	0.0293<br>(-0.032, 0.091)	0.032<br>(-0.058, 0.122)	0.061<br>(-0.012, 0.134)	0.00783
70	Sickle Cell Anemia (Hb-SS)	0.4<br>(0.37, 0.43)	0.59<br>(0.51, 0.68)	0.004<br>(0.0027, 0.0053)	0.7687<br>(0.675, 0.863)	-0.052<br>(-0.191, 0.086)	0.716<br>(0.604, 0.828)	0.00000
71	Thalassemia Major	0.12<br>(0.1, 0.14)	0.08<br>(-0.04, 0.21)	0.0213<br>(0.0195, 0.0231)	0.0483<br>(-0.153, 0.249)	-0.182<br>(-1.103, 0.739)	-0.134<br>(-0.907, 0.64)	0.70676
73	Combined and Other Severe Immunodeficiencies	0.16<br>(0.14, 0.18)	0.46<br>(0.39, 0.53)	0.0065<br>(0.0055, 0.0076)	0.0556<br>(-0.024, 0.135)	-0.127<br>(-0.243, -0.01)	-0.071<br>(-0.166, 0.023)	0.00141
74	Disorders of the Immune Mechanism	3.43<br>(3.33, 3.53)	0.37<br>(0.31, 0.43)	0.008<br>(0.0071, 0.0089)	0.0378<br>(-0.031, 0.107)	0.132<br>(0.03, 0.233)	0.17<br>(0.087, 0.252)	0.00000
75	Coagulation Defects and Other Specified Hematological Disorders	6.99<br>(6.85, 7.13)	0.52<br>(0.47, 0.58)	0.0063<br>(0.0055, 0.0071)	-0.0179<br>(-0.075, 0.039)	-0.083<br>(-0.166, 0.001)	-0.101<br>(-0.168, -0.033)	0.00001
81	Drug Psychosis	1.11<br>(1.06, 1.16)	0.4<br>(0.36, 0.45)	0.0069<br>(0.0062, 0.0075)	-0.7676<br>(-0.814, -0.721)	-0.156<br>(-0.224, -0.088)	-0.924<br>(-0.979, -0.868)	0.00000
82	Drug Dependence	9.93<br>(9.77, 10.09)	0.38<br>(0.33, 0.43)	0.0098<br>(0.0091, 0.0105)	-0.1235<br>(-0.177, -0.07)	-0.215<br>(-0.294, -0.136)	-0.338<br>(-0.402, -0.275)	0.00000
87	Schizophrenia	2.62<br>(2.54, 2.7)	0.78<br>(0.74, 0.82)	0.0031<br>(0.0024, 0.0037)	-0.0329<br>(-0.08, 0.014)	-0.031<br>(-0.1, 0.039)	-0.063<br>(-0.12, -0.007)	0.00018
88	Major Depressive and Bipolar Disorders	94.78<br>(94.27, 95.29)	0.89<br>(0.86, 0.92)	0.0009<br>(0.0005, 0.0014)	-0.5964<br>(-0.628, -0.565)	0.029<br>(-0.018, 0.075)	-0.568<br>(-0.605, -0.53)	0.00000

© 2020 Ellis RP et al. *JAMA Network Open.*

HCC	HCC Label	9/15 Rate	α	β	γ	δ	$\gamma+\delta$	F: γ=δ=0
89	Reactive and Unspecified Psychosis, Delusional Disorders	1.89 (1.82, 1.96)	0.63 (0.6, 0.66)	0.0055 (0.0051, 0.006)	-0.3593 (-0.394, -0.324)	-0.14 (-0.192, -0.089)	-0.500 (-0.542, -0.458)	0.00000
90	Personality Disorders	3.15 (3.06, 3.24)	0.49 (0.44, 0.55)	0.0063 (0.0055, 0.007)	-0.1646 (-0.222, -0.107)	-0.034 (-0.119, 0.051)	-0.198 (-0.267, -0.129)	0.00000
94	Anorexia/Bulimia Nervosa	1.74 (1.67, 1.81)	0.58 (0.53, 0.62)	0.005 (0.0043, 0.0056)	-0.0412 (-0.09, 0.007)	-0.036 (-0.107, 0.036)	-0.077 (-0.135, -0.019)	0.00001
96	Prader-Willi, Patau, Edwards, and Autosomal Deletion Syndromes	0.33 (0.3, 0.36)	0.37 (0.31, 0.43)	0.0064 (0.0055, 0.0073)	-0.0992 (-0.167, -0.031)	0.045 (-0.055, 0.146)	-0.054 (-0.135, 0.028)	0.00000
97	Down Syndrome, Fragile X, Other Chromosomal Anomalies, and Congenital Malformation Syndromes	2.19 (2.11, 2.27)	0.48 (0.43, 0.53)	0.0066 (0.0059, 0.0073)	0.036 (-0.017, 0.09)	-0.016 (-0.095, 0.063)	0.02 (-0.044, 0.084)	0.05132
102	Autistic Disorder	6.82 (6.68, 6.96)	0.18 (0.11, 0.26)	0.0098 (0.0086, 0.0109)	0.1801 (0.096, 0.265)	0.206 (0.082, 0.33)	0.386 (0.286, 0.487)	0.00000
103	Pervasive Developmental Disorders, Except Autistic Disorder	2.35 (2.27, 2.43)	1.18 (1.01, 1.34)	0.001 (-0.0015, 0.0034)	-0.5079 (-0.69, -0.326)	-0.067 (-0.334, 0.201)	-0.574 (-0.792, -0.357)	0.00000
106	Traumatic Complete Lesion Cervical Spinal Cord	0.01 (0, 0.02)	0.67 (0.42, 0.92)	0.0003 (-0.0035, 0.0041)	-0.4101 (-0.691, -0.129)	-0.125 (-0.539, 0.288)	-0.535 (-0.871, -0.2)	0.00000
107	Quadriplegia	0.38 (0.35, 0.41)	0.52 (0.46, 0.59)	0.0046 (0.0036, 0.0056)	0.0744 (0.001, 0.148)	-0.086 (-0.193, 0.022)	-0.011 (-0.099, 0.076)	0.00228
108	Traumatic Complete Lesion Dorsal Spinal Cord	0.01 (0, 0.02)	0.65 (0.37, 0.94)	0.0028 (-0.0014, 0.0071)	-0.2384 (-0.554, 0.078)	-0.427 (-0.892, 0.038)	-0.666 (-1.043, -0.289)	0.00000
109	Paraplegia	0.45 (0.42, 0.48)	0.55 (0.48, 0.62)	0.005 (0.004, 0.0061)	0.0744 (-0.001, 0.15)	-0.087 (-0.197, 0.024)	-0.012 (-0.102, 0.078)	0.00306
110	Spinal Cord Disorders/Injuries	1.15 (1.09, 1.21)	0.62 (0.56, 0.67)	0.0046 (0.0038, 0.0055)	0.1253 (0.062, 0.189)	-0.003 (-0.096, 0.091)	0.123 (0.047, 0.199)	0.00000
111	Amyotrophic Lateral Sclerosis and Other Anterior Horn Cell Disease	0.27 (0.24, 0.3)	0.74 (0.69, 0.8)	0.0027 (0.0019, 0.0036)	0.02 (-0.042, 0.081)	0.045 (-0.046, 0.135)	0.065 (-0.009, 0.138)	0.00917
112	Quadriplegic Cerebral Palsy	0.33 (0.3, 0.36)	0.75 (0.68, 0.81)	0.0027 (0.0018, 0.0037)	0.0612 (-0.01, 0.133)	0.244 (0.139, 0.349)	0.305 (0.22, 0.39)	0.00000
113	Cerebral Palsy, Except Quadriplegic	1.76 (1.69, 1.83)	0.66 (0.6, 0.71)	0.0039 (0.0031, 0.0047)	0.0772 (0.017, 0.138)	-0.023 (-0.113, 0.066)	0.054 (-0.019, 0.126)	0.00003
114	Spina Bifida and Other Brain/Spinal/Nervous System Congenital Anomalies	1.28 (1.22, 1.34)	0.6 (0.55, 0.66)	0.0049 (0.004, 0.0057)	0.0299 (-0.033, 0.093)	-0.061 (-0.154, 0.032)	-0.031 (-0.107, 0.044)	0.07419

© 2020 Ellis RP et al. *JAMA Network Open.*

HCC	HCC Label	9/15 Rate	α	β	γ	δ	$\gamma+\delta$	F: $\gamma=\delta=0$
115	Myasthenia Gravis/Myoneural Disorders and Guillain-Barre Syndrome/Inflammatory and Toxic Neuropathy	2.2 (2.12, 2.28)	0.57 (0.51, 0.62)	0.0048 (0.004, 0.0056)	0.025 (-0.034, 0.084)	0.013 (-0.073, 0.1)	0.038 (-0.032, 0.108)	0.07995
117	Muscular Dystrophy	0.38 (0.35, 0.41)	0.63 (0.57, 0.69)	0.0036 (0.0027, 0.0046)	0.1299 (0.059, 0.201)	-0.018 (-0.123, 0.086)	0.112 (0.027, 0.196)	0.00000
118	Multiple Sclerosis	5.68 (5.56, 5.8)	0.83 (0.79, 0.88)	0.0018 (0.0012, 0.0025)	0.0081 (-0.042, 0.058)	-0.012 (-0.085, 0.061)	-0.004 (-0.063, 0.055)	0.81609
119	Parkinson`s, Huntington`s, and Spinocerebellar Disease, and Other Neurodegenerative Disorders	1.63 (1.56, 1.7)	0.69 (0.64, 0.74)	0.0034 (0.0026, 0.0041)	-0.0109 (-0.067, 0.045)	-0.056 (-0.138, 0.026)	-0.067 (-0.133, 0)	0.00348
120	Seizure Disorders and Convulsions	12.16 (11.98, 12.34)	0.76 (0.72, 0.81)	0.0033 (0.0026, 0.004)	-0.0254 (-0.077, 0.026)	-0.043 (-0.119, 0.032)	-0.069 (-0.13, -0.007)	0.00048
121	Hydrocephalus	0.89 (0.84, 0.94)	0.58 (0.52, 0.63)	0.0051 (0.0043, 0.006)	-0.1068 (-0.171, -0.043)	-0.087 (-0.181, 0.007)	-0.194 (-0.27, -0.118)	0.00000
122	Non-Traumatic Coma, Brain Compression/Anoxic Damage	0.78 (0.73, 0.83)	0.5 (0.44, 0.57)	0.0065 (0.0055, 0.0075)	0.1205 (0.048, 0.193)	0.194 (0.087, 0.3)	0.314 (0.228, 0.401)	0.00000
125	Respirator Dependence/Tracheostomy Status	0.4 (0.37, 0.43)	0.42 (0.35, 0.49)	0.0073 (0.0063, 0.0083)	0.0613 (-0.012, 0.135)	-0.087 (-0.196, 0.021)	-0.026 (-0.114, 0.062)	0.00848
126	Respiratory Arrest	0.05 (0.04, 0.06)	0.93 (0.78, 1.09)	-0.0005 (-0.0028, 0.0018)	0.0939 (-0.079, 0.267)	-0.299 (-0.553, -0.045)	-0.205 (-0.411, 0.001)	0.00042
127	Cardio-Respiratory Failure and Shock, Including Respiratory Distress Syndromes	3.78 (3.68, 3.88)	0.8 (0.75, 0.85)	0.0035 (0.0027, 0.0042)	0.0017 (-0.053, 0.057)	-0.086 (-0.167, -0.006)	-0.085 (-0.15, -0.019)	0.00011
128	Heart Assistive Device/Artificial Heart	0.09 (0.07, 0.11)	0.18 (0.1, 0.27)	0.008 (0.0067, 0.0092)	0.2015 (0.108, 0.295)	-0.118 (-0.255, 0.02)	0.084 (-0.027, 0.195)	0.00000
129	Heart Transplant	0.23 (0.21, 0.25)	0.81 (0.74, 0.88)	0.0024 (0.0013, 0.0035)	0.04 (-0.042, 0.122)	0.033 (-0.088, 0.154)	0.073 (-0.025, 0.171)	0.01603
130	Congestive Heart Failure	11.82 (11.64, 12)	0.68 (0.62, 0.74)	0.004 (0.0031, 0.0048)	0.0435 (-0.019, 0.106)	-0.056 (-0.149, 0.036)	-0.013 (-0.088, 0.062)	0.04424
131	Acute Myocardial Infarction	0.88 (0.83, 0.93)	0.82 (0.76, 0.89)	0.0016 (0.0006, 0.0026)	1.3145 (1.241, 1.388)	0.159 (0.051, 0.267)	1.474 (1.386, 1.561)	0.00000
132	Unstable Angina and Other Acute Ischemic Heart Disease	2.85 (2.76, 2.94)	0.81 (0.76, 0.86)	0.0017 (0.0009, 0.0025)	-0.316 (-0.375, -0.257)	-0.075 (-0.161, 0.012)	-0.391 (-0.461, -0.32)	0.00000
135	Heart Infection/Inflammation, Except Rheumatic	1.05 (1, 1.1)	0.64 (0.58, 0.7)	0.0049 (0.0041, 0.0058)	0.0887 (0.027, 0.15)	-0.025 (-0.116, 0.065)	0.063 (-0.01, 0.137)	0.00000
137	Hypoplastic Left Heart Syndrome and Other Severe Congenital Heart Disorders	0.21 (0.19, 0.23)	0.63 (0.54, 0.71)	0.0034 (0.0021, 0.0047)	0.1937 (0.096, 0.291)	0.127 (-0.017, 0.27)	0.32 (0.204, 0.437)	0.00000

© 2020 Ellis RP et al. *JAMA Network Open.*

HCC	HCC Label	9/15 Rate	α	β	γ	δ	$\gamma+\delta$	F: $\gamma=\delta=0$
138	Major Congenital Heart/Circulatory Disorders	2.56 (2.48, 2.64)	0.63 (0.58, 0.69)	0.0046 (0.0038, 0.0054)	-0.0251 (-0.085, 0.034)	-0.008 (-0.095, 0.08)	-0.033 (-0.104, 0.038)	0.12699
139	Atrial and Ventricular Septal Defects, Patent Ductus Arteriosus, and Other Congenital Heart/Circulatory Disorders	3.13 (3.04, 3.22)	0.66 (0.61, 0.72)	0.0039 (0.0031, 0.0047)	0.0411 (-0.017, 0.099)	-0.003 (-0.089, 0.083)	0.038 (-0.032, 0.108)	0.01614
142	Specified Heart Arrhythmias	16.7 (16.49, 16.91)	0.74 (0.69, 0.79)	0.003 (0.0023, 0.0038)	0.0611 (0.005, 0.117)	-0.024 (-0.107, 0.059)	0.037 (-0.03, 0.104)	0.00057
145	Intracranial Hemorrhage	0.95 (0.9, 1)	0.57 (0.53, 0.61)	0.0049 (0.0043, 0.0055)	-0.0196 (-0.065, 0.025)	-0.061 (-0.127, 0.005)	-0.081 (-0.134, -0.027)	0.00001
146	Ischemic or Unspecified Stroke	2.93 (2.84, 3.02)	0.45 (0.38, 0.51)	0.0064 (0.0054, 0.0074)	0.1099 (0.036, 0.183)	-0.054 (-0.162, 0.054)	0.056 (-0.032, 0.144)	0.00000
149	Cerebral Aneurysm and Arteriovenous Malformation	0.62 (0.58, 0.66)	0.63 (0.56, 0.69)	0.0051 (0.0041, 0.0061)	0.0533 (-0.02, 0.126)	0.028 (-0.08, 0.135)	0.081 (-0.006, 0.168)	0.00097
150	Hemiplegia/Hemiparesis	1.47 (1.41, 1.53)	0.55 (0.5, 0.6)	0.0053 (0.0045, 0.006)	0.1426 (0.086, 0.2)	-0.083 (-0.167, 0.001)	0.059 (-0.009, 0.128)	0.00000
151	Monoplegia, Other Paralytic Syndromes	0.24 (0.21, 0.27)	0.65 (0.56, 0.73)	0.0044 (0.0031, 0.0057)	0.1304 (0.034, 0.227)	0.021 (-0.121, 0.163)	0.151 (0.036, 0.267)	0.00000
153	Atherosclerosis of the Extremities with Ulceration or Gangrene	0.4 (0.37, 0.43)	0.76 (0.68, 0.83)	0.0037 (0.0026, 0.0048)	0.184 (0.105, 0.263)	-0.06 (-0.177, 0.056)	0.124 (0.029, 0.218)	0.00000
154	Vascular Disease with Complications	0.83 (0.78, 0.88)	0.7 (0.65, 0.75)	0.0043 (0.0036, 0.005)	-0.1412 (-0.195, -0.088)	0.017 (-0.061, 0.096)	-0.124 (-0.188, -0.06)	0.00000
156	Pulmonary Embolism and Deep Vein Thrombosis	7.05 (6.91, 7.19)	0.63 (0.59, 0.67)	0.0051 (0.0046, 0.0057)	-0.0674 (-0.108, -0.027)	-0.155 (-0.214, -0.095)	-0.222 (-0.271, -0.173)	0.00000
158	Lung Transplant Status/Complications	0.13 (0.11, 0.15)	0.77 (0.67, 0.87)	0.0044 (0.0029, 0.0059)	-0.1099 (-0.222, 0.002)	0.17 (0.005, 0.335)	0.06 (-0.074, 0.194)	0.00089
159	Cystic Fibrosis	0.58 (0.54, 0.62)	0.85 (0.79, 0.91)	0.0021 (0.0012, 0.0029)	-0.0137 (-0.078, 0.051)	0.043 (-0.052, 0.137)	0.029 (-0.047, 0.106)	0.28577
160	Chronic Obstructive Pulmonary Disease, Including Bronchiectasis	13.44 (13.25, 13.63)	0.92 (0.85, 0.99)	0.0021 (0.001, 0.0031)	-0.0459 (-0.124, 0.033)	-0.112 (-0.228, 0.003)	-0.158 (-0.252, -0.065)	0.00000
161	Asthma	67.61 (67.18, 68.04)	0.81 (0.75, 0.86)	0.0034 (0.0025, 0.0043)	0.0047 (-0.06, 0.069)	-0.063 (-0.158, 0.031)	-0.059 (-0.135, 0.018)	0.02945
162	Fibrosis of Lung and Other Lung Disorders	3.8 (3.7, 3.9)	0.61 (0.55, 0.67)	0.0052 (0.0043, 0.0061)	-0.4071 (-0.475, -0.339)	-0.114 (-0.214, -0.015)	-0.521 (-0.602, -0.441)	0.00000
163	Aspiration and Specified Bacterial Pneumonias and Other Severe Lung Infections	0.82 (0.77, 0.87)	1.01 (0.94, 1.08)	0.0006 (-0.0005, 0.0017)	0.2025 (0.12, 0.285)	-0.044 (-0.165, 0.077)	0.158 (0.06, 0.256)	0.00000
183	Kidney Transplant Status	1.6 (1.53, 1.67)	0.69 (0.64, 0.74)	0.004 (0.0032, 0.0047)	0.0635 (0.007, 0.12)	-0.025 (-0.109, 0.059)	0.038 (-0.03, 0.106)	0.00038

© 2020 Ellis RP et al. *JAMA Network Open.*

HCC	HCC Label	9/15 Rate	α	β	γ	δ	$\gamma + \delta$	F: γ=δ=0
184	End Stage Renal Disease	4.69 (4.58, 4.8)	0.77 (0.72, 0.81)	0.002 (0.0012, 0.0027)	0.0595 (0.006, 0.113)	-0.064 (-0.142, 0.014)	-0.004 (-0.068, 0.059)	0.00082
187	Chronic Kidney Disease, Stage 5	1.38 (1.32, 1.44)	0.65 (0.58, 0.72)	0.0036 (0.0026, 0.0046)	0.1061 (0.031, 0.181)	0.112 (0.002, 0.222)	0.218 (0.129, 0.307)	0.00000
188	Chronic Kidney Disease, Severe (Stage 4)	1.39 (1.33, 1.45)	0.67 (0.6, 0.74)	0.0037 (0.0027, 0.0047)	0.0812 (0.005, 0.157)	-0.071 (-0.183, 0.04)	0.01 (-0.081, 0.1)	0.00171
203	Ectopic and Molar Pregnancy, Except with Renal Failure, Shock, or Embolism	0.39 (0.36, 0.42)	0.93 (0.85, 1.01)	0.0012 (-0.0001, 0.0024)	-0.9029 (-0.996, -0.81)	0.460 (0.323, 0.596)	-0.443 (-0.554, -0.333)	0.00000
204	Miscarriage with Complications	0.08 (0.07, 0.09)	1.42 (1.3, 1.54)	-0.0052 (-0.007, -0.0034)	-0.0675 (-0.203, 0.068)	0.015 (-0.185, 0.214)	-0.053 (-0.214, 0.109)	0.15033
205	Miscarriage with No or Minor Complications	2.23 (2.15, 2.31)	0.95 (0.9, 1)	0.0011 (0.0003, 0.0018)	-0.017 (-0.071, 0.037)	-0.004 (-0.084, 0.075)	-0.021 (-0.086, 0.043)	0.33292
207	Completed Pregnancy With Major Complications	0.4 (0.37, 0.43)	0.56 (0.48, 0.64)	0.0045 (0.0033, 0.0058)	-0.4459 (-0.538, -0.354)	0.195 (0.06, 0.33)	-0.251 (-0.361, -0.141)	0.00000
208	Completed Pregnancy With Complications	5.17 (5.05, 5.29)	0.68 (0.64, 0.72)	0.0027 (0.0021, 0.0032)	-0.5448 (-0.587, -0.502)	0.037 (-0.026, 0.099)	-0.508 (-0.559, -0.457)	0.00000
209	Completed Pregnancy with No or Minor Complications	12.4 (12.22, 12.58)	0.72 (0.69, 0.75)	0.0021 (0.0017, 0.0025)	0.0287 (-0.002, 0.059)	-0.033 (-0.078, 0.011)	-0.004 (-0.04, 0.032)	0.00476
217	Chronic Ulcer of Skin, Except Pressure	5.24 (5.12, 5.36)	0.71 (0.66, 0.76)	0.0028 (0.0021, 0.0035)	-0.1054 (-0.159, -0.052)	-0.034 (-0.113, 0.044)	-0.14 (-0.204, -0.076)	0.00000
226	Hip Fractures and Pathological Vertebral or Humerus Fractures	0.65 (0.61, 0.69)	0.89 (0.83, 0.94)	0.0014 (0.0006, 0.0022)	-0.3464 (-0.407, -0.285)	0.072 (-0.017, 0.162)	-0.274 (-0.347, -0.201)	0.00000
227	Pathological Fractures, Except of Vertebrae, Hip, or Humerus	0.26 (0.23, 0.29)	0.93 (0.84, 1.02)	-0.0018 (-0.0031, -0.0005)	-0.1539 (-0.253, -0.055)	-0.028 (-0.174, 0.117)	-0.182 (-0.3, -0.064)	0.00000
242	Extremely Immature Newborns, Birthweight < 500 Grams	0.01 (0, 0.02)	1.12 (0.78, 1.45)	0.0009 (-0.004, 0.0059)	-0.4966 (-0.865, -0.128)	-0.076 (-0.618, 0.467)	-0.572 (-1.013, -0.132)	0.00000
243	Extremely Immature Newborns, Including Birthweight 500-749 Grams	0.08 (0.07, 0.09)	0.69 (0.6, 0.79)	0.004 (0.0026, 0.0055)	-0.128 (-0.236, -0.02)	-0.034 (-0.193, 0.126)	-0.162 (-0.291, -0.033)	0.00000
244	Extremely Immature Newborns, Including Birthweight 750-999 Grams	0.1 (0.08, 0.12)	0.92 (0.82, 1.01)	0.002 (0.0006, 0.0035)	-0.0837 (-0.194, 0.026)	-0.101 (-0.263, 0.061)	-0.185 (-0.316, -0.054)	0.00001
245	Premature Newborns, Including Birthweight 1000-1499 Grams	0.18 (0.16, 0.2)	0.91 (0.82, 1)	0.0029 (0.0015, 0.0043)	-0.0596 (-0.161, 0.042)	-0.020 (-0.17, 0.129)	-0.08 (-0.201, 0.041)	0.01809
246	Premature Newborns, Including Birthweight 1500-1999 Grams	0.28 (0.25, 0.31)	1.04 (0.93, 1.16)	0.0014 (-0.0003, 0.0031)	-0.1113 (-0.238, 0.016)	0.229 (0.043, 0.416)	0.118 (-0.033, 0.27)	0.00024
247	Premature Newborns, Including Birthweight 2000-2499 Grams	0.35 (0.32, 0.38)	0.82 (0.74, 0.9)	0.0026 (0.0014, 0.0039)	-0.0324 (-0.124, 0.059)	0.186 (0.051, 0.321)	0.154 (0.045, 0.263)	0.00001
248	Other Premature, Low Birthweight, Malnourished, or Multiple Birth Newborns	1 (0.95, 1.05)	0.79 (0.72, 0.86)	0.0026 (0.0015, 0.0036)	-0.1072 (-0.184, -0.03)	0.063 (-0.05, 0.176)	-0.045 (-0.136, 0.047)	0.00002

© 2020 Ellis RP et al. *JAMA Network Open.*

HCC	HCC Label	9/15 Rate	α	β	γ	δ	$\gamma + \delta$	F: γ=δ=0
249	Term or Post-Term Singleton Newborn, Normal or High Birthweight	7.28 (7.14, 7.42)	0.95 (0.91, 0.99)	-0.0012 (-0.0019, -0.0006)	-0.0150 (-0.063, 0.033)	0.030 (-0.04, 0.101)	0.015 (-0.042, 0.072)	0.32129
251	Stem Cell, Including Bone Marrow, Transplant Status/Complications	0.62 (0.58, 0.66)	0.4 (0.35, 0.45)	0.0084 (0.0076, 0.0091)	0.009 (-0.044, 0.062)	0.031 (-0.047, 0.109)	0.04 (-0.023, 0.103)	0.08934
253	Artificial Openings for Feeding or Elimination	1.34 (1.28, 1.4)	0.53 (0.48, 0.59)	0.006 (0.0052, 0.0068)	0.0392 (-0.019, 0.097)	0.028 (-0.057, 0.114)	0.068 (-0.002, 0.137)	0.00092
254	Amputation Status, Lower Limb/Amputation Complications	0.51 (0.47, 0.55)	0.38 (0.31, 0.45)	0.0073 (0.0062, 0.0084)	0.2325 (0.15, 0.314)	0.04 (-0.081, 0.161)	0.273 (0.175, 0.37)	0.00000
ALL	# of categories (out of 127), where coefficients are statistically significant (that is, P < 0.05/127)		126	116	56	31	70	75
ALL	# of categories with coefficients that are statistically significant and large (|coef| ≥20%)		-	-	20	12	30	-

© 2020 Ellis RP et al. *JAMA Network Open.*

eTable 3. Piecewise Linear Regression Coefficients Predicting 2010 to 2017 Monthly Prevalence for 282 Agency for Healthcare Research and Quality Clinical Classification System Categories

Gray-shaded regions are statistically significant; darker (blue) shaded areas are statistically significant and large, that is, |coefficient| ≥ 0.2.
Confidence intervals (CI) and statistical significance calculated using the Bonferroni correction.

		Sept 2015 prevalence per 10,000 enrollees	Intercept: Estimated relative rate in Dec 2009 (CI)	Slope: Estimated trend rate before ICD-10 (CI)	Level change in October 2015 (CI)	Time trend change Oct 2015 – Dec 2017 (CI)	Cumulative effect of level and time trend changes (CI)	F-test: For a straight line trend (p-value)
**CCS**	**Category label**	**9/15 Rate**	α	β	γ	δ	$\gamma + \delta$	**F:** $\gamma=\delta=0$
1	Tuberculosis	0.22 (0.2, 0.24)	0.91 (0.81, 1.01)	0.0011 (-0.0003, 0.0026)	-0.0353 (-0.141, 0.071)	-0.131 (-0.287, 0.025)	-0.167 (-0.293, -0.04)	0.00002
2	Septicemia (Except in Labor)	2.64 (2.56, 2.72)	0.54 (0.48, 0.59)	0.0054 (0.0046, 0.0062)	-0.0405 (-0.099, 0.018)	-0.08 (-0.166, 0.006)	-0.12 (-0.19, -0.05)	0
3	Bacterial Infection; Unspecified Site	8.27 (8.12, 8.42)	0.48 (0.36, 0.6)	0.0068 (0.0051, 0.0085)	0.3416 (0.214, 0.470)	0.151 (-0.037, 0.34)	0.493 (0.34, 0.646)	0
4	Mycoses	39.68 (39.35, 40.01)	0.56 (0.5, 0.62)	0.0043 (0.0034, 0.0051)	0.0678 (0.002, 0.134)	-0.127 (-0.224, -0.03)	-0.059 (-0.137, 0.02)	0.00002
5	HIV Infection	3.81 (3.71, 3.91)	0.81 (0.75, 0.87)	0.0021 (0.0012, 0.003)	0.0201 (-0.048, 0.089)	0.101 (0, 0.202)	0.121 (0.039, 0.203)	0
6	Hepatitis	5.23 (5.11, 5.35)	0.8 (0.73, 0.88)	0.0026 (0.0015, 0.0037)	0.0045 (-0.078, 0.087)	-0.124 (-0.246, -0.003)	-0.120 (-0.218, -0.021)	0.0001
7	Viral Infection	64.92 (64.5, 65.34)	1.01 (0.89, 1.12)	0.0017 (0, 0.0034)	-0.0314 (-0.157, 0.095)	0.124 (-0.061, 0.309)	0.093 (-0.058, 0.243)	0.04152
8	Other Infections; Including Parasitic	7.95 (7.8, 8.1)	0.52 (0.46, 0.57)	0.0049 (0.0041, 0.0057)	-0.1895 (-0.252, -0.127)	-0.096 (-0.188, -0.004)	-0.286 (-0.36, -0.211)	0
9	Sexually Transmitted Infections (Not HIV or Hepatitis)	3.7 (3.6, 3.8)	0.63 (0.57, 0.68)	0.005 (0.0042, 0.0058)	0.0098 (-0.049, 0.069)	0.019 (-0.068, 0.106)	0.029 (-0.042, 0.099)	0.29889
10	Immunizations and Screening for Infectious Disease	187.47 (186.76, 188.18)	0.21 (0.08, 0.34)	0.006 (0.004, 0.0079)	0.1689 (0.024, 0.314)	-0.011 (-0.225, 0.202)	0.157 (-0.015, 0.33)	0.00001
11	Cancer of Head and Neck	1.9 (1.83, 1.97)	0.87 (0.83, 0.91)	0.0016 (0.0009, 0.0022)	-0.028 (-0.074, 0.018)	-0.038 (-0.106, 0.03)	-0.066 (-0.121, -0.011)	0.00004
12	Cancer of Esophagus	0.4 (0.37, 0.43)	0.86 (0.81, 0.91)	0.0007 (-0.0001, 0.0014)	-0.0137 (-0.069, 0.042)	0.033 (-0.049, 0.115)	0.019 (-0.047, 0.085)	0.33923
13	Cancer of Stomach	0.35 (0.32, 0.38)	0.84 (0.78, 0.9)	0.0014 (0.0005, 0.0023)	-0.05 (-0.116, 0.016)	0.263 (0.166, 0.359)	0.213 (0.134, 0.291)	0

© 2020 Ellis RP et al. *JAMA Network Open.*

CCS	Category label	9/15 Rate	α	β	γ	δ	$\gamma+\delta$	F: $\gamma=\delta=0$
14	Cancer of Colon	3.17 (3.08, 3.26)	0.83 (0.79, 0.87)	0.0013 (0.0007, 0.0019)	-0.0123 (-0.058, 0.033)	-0.022 (-0.088, 0.045)	-0.034 (-0.088, 0.02)	0.05427
15	Cancer of Rectum and Anus	1.76 (1.69, 1.83)	0.86 (0.81, 0.9)	0.0008 (0.0001, 0.0015)	0.0417 (-0.008, 0.092)	0.058 (-0.015, 0.132)	0.100 (0.04, 0.16)	0
16	Cancer of Liver and Intrahepatic Bile Duct	0.46 (0.42, 0.5)	0.7 (0.66, 0.75)	0.0038 (0.0031, 0.0045)	-0.0474 (-0.099, 0.004)	-0.078 (-0.154, -0.002)	-0.125 (-0.187, -0.064)	0
17	Cancer of Pancreas	0.65 (0.61, 0.69)	0.75 (0.72, 0.78)	0.0031 (0.0027, 0.0036)	-0.0364 (-0.069, -0.004)	-0.045 (-0.093, 0.003)	-0.081 (-0.12, -0.042)	0
18	Cancer of Other GI Organs; Peritoneum	0.62 (0.58, 0.66)	0.66 (0.61, 0.7)	0.0039 (0.0032, 0.0046)	0.0033 (-0.046, 0.053)	0.019 (-0.053, 0.092)	0.023 (-0.036, 0.082)	0.36677
19	Cancer of Bronchus; Lung	2.22 (2.14, 2.3)	0.97 (0.93, 1.01)	-0.0003 (-0.0009, 0.0003)	-0.0137 (-0.058, 0.031)	0.032 (-0.034, 0.098)	0.018 (-0.035, 0.071)	0.20684
20	Cancer; Other Respiratory and Intrathoracic	0.07 (0.06, 0.08)	0.86 (0.74, 0.99)	0.0001 (-0.0017, 0.002)	-0.0717 (-0.209, 0.066)	0.026 (-0.176, 0.228)	-0.046 (-0.21, 0.118)	0.11511
21	Cancer of Bone and Connective Tissue	0.92 (0.87, 0.97)	0.87 (0.83, 0.92)	0.0015 (0.0008, 0.0021)	-0.016 (-0.064, 0.032)	0.008 (-0.063, 0.079)	-0.008 (-0.065, 0.049)	0.43664
22	Melanomas of Skin	4.18 (4.07, 4.29)	0.56 (0.5, 0.62)	0.0056 (0.0047, 0.0065)	-0.1176 (-0.186, -0.049)	-0.091 (-0.192, 0.01)	-0.209 (-0.29, -0.127)	0
23	Other Non-Epithelial Cancer of Skin	14.89 (14.69, 15.09)	0.62 (0.55, 0.69)	0.0041 (0.0031, 0.0051)	-0.0521 (-0.13, 0.026)	-0.109 (-0.223, 0.005)	-0.161 (-0.254, -0.068)	0
24	Cancer of Breast	18.71 (18.48, 18.94)	0.81 (0.77, 0.86)	0.0017 (0.001, 0.0023)	-0.005 (-0.054, 0.044)	0 (-0.071, 0.072)	-0.005 (-0.063, 0.053)	0.89643
25	Cancer of Uterus	1.44 (1.38, 1.5)	0.72 (0.67, 0.77)	0.0036 (0.0029, 0.0043)	-0.0308 (-0.083, 0.021)	-0.018 (-0.094, 0.058)	-0.049 (-0.11, 0.013)	0.00338
26	Cancer of Cervix	5.74 (5.62, 5.86)	1.38 (1.27, 1.48)	-0.0056 (-0.0073, -0.004)	0.0792 (-0.041, 0.2)	0.194 (0.017, 0.372)	0.273 (0.129, 0.417)	0
27	Cancer of Ovary	1.31 (1.25, 1.37)	0.91 (0.87, 0.95)	0.0008 (0.0002, 0.0014)	-0.0354 (-0.079, 0.008)	-0.016 (-0.08, 0.047)	-0.052 (-0.103, 0)	0.0001
28	Cancer of Other Female Genital Organs	0.4 (0.37, 0.43)	0.82 (0.74, 0.89)	0.0021 (0.001, 0.0032)	0.5476 (0.464, 0.631)	-0.071 (-0.195, 0.052)	0.476 (0.376, 0.576)	0
29	Cancer of Prostate	5.41 (5.29, 5.53)	0.89 (0.84, 0.95)	0.0005 (-0.0004, 0.0013)	0.003 (-0.059, 0.065)	0.042 (-0.049, 0.133)	0.045 (-0.029, 0.119)	0.08771
30	Cancer of Testis	0.62 (0.58, 0.66)	0.8 (0.74, 0.86)	0.0026 (0.0017, 0.0035)	0.0118 (-0.055, 0.079)	-0.04 (-0.138, 0.058)	-0.028 (-0.108, 0.051)	0.30929

© 2020 Ellis RP et al. *JAMA Network Open.*

CCS	Category label	9/15 Rate	α	β	γ	δ	$\gamma+\delta$	F: $\gamma=\delta=0$
31	Cancer of Other Male Genital Organs	0.03 (0.02, 0.04)	1.31 (1.07, 1.55)	-0.0004 (-0.0039, 0.0031)	0.2216 (-0.042, 0.485)	-0.06 (-0.447, 0.328)	0.162 (-0.153, 0.476)	0.00341
32	Cancer of Bladder	1.26 (1.2, 1.32)	0.92 (0.86, 0.97)	0.0006 (-0.0002, 0.0014)	-0.0204 (-0.078, 0.037)	-0.009 (-0.094, 0.076)	-0.029 (-0.098, 0.039)	0.15349
33	Cancer of Kidney and Renal Pelvis	1.3 (1.24, 1.36)	0.74 (0.69, 0.79)	0.0031 (0.0022, 0.0039)	0.0282 (-0.032, 0.088)	0.024 (-0.065, 0.112)	0.052 (-0.02, 0.123)	0.01215
34	Cancer of Other Urinary Organs	0.08 (0.07, 0.09)	0.88 (0.76, 1)	0.002 (0.0002, 0.0039)	-0.0967 (-0.232, 0.039)	0.209 (0.009, 0.408)	0.112 (-0.05, 0.274)	0.00094
35	Cancer of Brain and Nervous System	1.24 (1.18, 1.3)	0.84 (0.8, 0.88)	0.0014 (0.0008, 0.002)	0.0439 (-0.001, 0.089)	-0.079 (-0.145, -0.013)	-0.035 (-0.088, 0.019)	0.00009
36	Cancer of Thyroid	3.02 (2.93, 3.11)	0.59 (0.53, 0.64)	0.0055 (0.0047, 0.0063)	0.0154 (-0.044, 0.075)	-0.107 (-0.195, -0.02)	-0.092 (-0.163, -0.021)	0.00002
37	Hodgkin's Disease	0.79 (0.74, 0.84)	0.89 (0.84, 0.94)	0.0015 (0.0007, 0.0022)	0.0092 (-0.046, 0.064)	-0.001 (-0.081, 0.08)	0.009 (-0.057, 0.074)	0.75697
38	Non-Hodgkin's Lymphoma	2.89 (2.8, 2.98)	0.92 (0.88, 0.96)	0.0004 (-0.0002, 0.0011)	0.0007 (-0.046, 0.047)	0.029 (-0.04, 0.098)	0.03 (-0.026, 0.085)	0.14776
39	Leukemias	2.48 (2.4, 2.56)	0.83 (0.79, 0.87)	0.0024 (0.0018, 0.0029)	-0.0094 (-0.05, 0.031)	0.037 (-0.023, 0.097)	0.027 (-0.021, 0.076)	0.06955
40	Multiple Myeloma	1.18 (1.12, 1.24)	0.7 (0.67, 0.74)	0.0041 (0.0035, 0.0046)	0.0066 (-0.035, 0.048)	-0.008 (-0.069, 0.052)	-0.002 (-0.051, 0.047)	0.82527
41	Cancer; Other and Unspecified Primary	1.4 (1.34, 1.46)	0.87 (0.81, 0.93)	0.0017 (0.0007, 0.0026)	0.0148 (-0.053, 0.082)	0.311 (0.212, 0.41)	0.326 (0.245, 0.406)	0
42	Secondary Malignancies	6.08 (5.95, 6.21)	0.53 (0.49, 0.58)	0.0063 (0.0057, 0.007)	0.048 (-0.001, 0.097)	0.031 (-0.041, 0.103)	0.079 (0.021, 0.138)	0
43	Malignant Neoplasm Without Specification of Site	1.16 (1.1, 1.22)	0.37 (0.28, 0.45)	0.0094 (0.0082, 0.0107)	-0.14 (-0.232, -0.048)	-0.220 (-0.356, -0.084)	-0.360 (-0.47, -0.25)	0
44	Neoplasms of Unspecified Nature or Uncertain Behavior	39.66 (39.33, 39.99)	0.71 (0.65, 0.78)	0.0025 (0.0015, 0.0035)	-0.0447 (-0.117, 0.027)	-0.040 (-0.145, 0.066)	-0.085 (-0.17, 0.001)	0.00044
45	Maintenance Chemotherapy; Radiotherapy	8.87 (8.71, 9.03)	0.56 (0.51, 0.61)	0.0054 (0.0046, 0.0062)	-0.0144 (-0.072, 0.044)	-0.047 (-0.132, 0.039)	-0.061 (-0.13, 0.008)	0.00585

© 2020 Ellis RP et al. *JAMA Network Open.*

CCS	Category label	9/15 Rate	α	β	γ	δ	$\gamma+\delta$	F: $\gamma=\delta=0$
46	Benign Neoplasm of Uterus	8.36 (8.21, 8.51)	0.64 (0.57, 0.71)	0.0037 (0.0027, 0.0048)	0.0794 (0.004, 0.155)	-0.055 (-0.165, 0.056)	0.025 (-0.065, 0.115)	0.00079
47	Other and unspecified benign neoplasm	91.24 (90.74, 91.74)	0.53 (0.46, 0.6)	0.0047 (0.0036, 0.0058)	0.0462 (-0.034, 0.126)	-0.004 (-0.122, 0.113)	0.042 (-0.054, 0.137)	0.0433
48	Thyroid Disorders	87.33 (86.84, 87.82)	0.51 (0.45, 0.58)	0.0064 (0.0054, 0.0073)	-0.0197 (-0.088, 0.049)	-0.094 (-0.196, 0.007)	-0.114 (-0.196, -0.032)	0.00001
49	Diabetes Mellitus Without Complication	118.99 (118.42, 119.56)	0.57 (0.5, 0.64)	0.0053 (0.0043, 0.0063)	0.0286 (-0.045, 0.102)	-0.105 (-0.213, 0.003)	-0.076 (-0.164, 0.011)	0.00176
50	Diabetes Mellitus with Complications	51.16 (50.79, 51.53)	0.66 (0.6, 0.72)	0.0037 (0.0028, 0.0046)	0.1385 (0.072, 0.205)	0.053 (-0.045, 0.151)	0.192 (0.112, 0.271)	0
51	Other Endocrine Disorders	39 (38.68, 39.32)	0.43 (0.38, 0.48)	0.0085 (0.0078, 0.0092)	-0.0606 (-0.112, -0.009)	-0.114 (-0.19, -0.038)	-0.174 (-0.236, -0.113)	0
52	Nutritional Deficiencies	46.62 (46.26, 46.98)	0.21 (0.13, 0.3)	0.0102 (0.009, 0.0114)	0.135 (0.045, 0.225)	-0.001 (-0.134, 0.131)	0.134 (0.027, 0.241)	0
53	Disorders of Lipid Metabolism	187.56 (186.85, 188.27)	0.58 (0.51, 0.65)	0.0056 (0.0046, 0.0067)	0.0108 (-0.068, 0.089)	-0.104 (-0.22, 0.012)	-0.093 (-0.187, 0.001)	0.00137
54	Gout and Other Crystal Arthropathies	10.32 (10.15, 10.49)	0.53 (0.47, 0.59)	0.006 (0.0051, 0.0069)	-0.0667 (-0.133, -0.001)	-0.124 (-0.221, -0.027)	-0.191 (-0.27, -0.112)	0
55	Fluid and Electrolyte Disorders	16.94 (16.73, 17.15)	0.56 (0.51, 0.62)	0.0064 (0.0055, 0.0072)	-0.0769 (-0.139, -0.014)	-0.039 (-0.131, 0.053)	-0.116 (-0.19, -0.041)	0
56	Cystic Fibrosis	0.58 (0.54, 0.62)	0.85 (0.79, 0.91)	0.0021 (0.0012, 0.003)	-0.0137 (-0.082, 0.055)	0.043 (-0.058, 0.144)	0.029 (-0.053, 0.111)	0.28577
57	Immunity Disorders	3.41 (3.31, 3.51)	0.36 (0.29, 0.44)	0.008 (0.0069, 0.0092)	0.616 (0.534, 0.699)	0.164 (0.043, 0.285)	0.780 (0.682, 0.879)	0
58	Other Nutritional; Endocrine; and Metabolic Disorders	157.67 (157.02, 158.32)	0.19 (0.08, 0.3)	0.0097 (0.0082, 0.0113)	0.1691 (0.051, 0.287)	0.257 (0.084, 0.431)	0.426 (0.285, 0.567)	0
59	Deficiency and Other Anemia	37.26 (36.94, 37.58)	0.57 (0.51, 0.63)	0.005 (0.0041, 0.006)	0.0299 (-0.037, 0.097)	-0.043 (-0.142, 0.056)	-0.013 (-0.093, 0.067)	0.19192
60	Acute Posthemorrhagic Anemia	1.11 (1.06, 1.16)	0.47 (0.41, 0.54)	0.0065 (0.0056, 0.0075)	0.0699 (-0.002, 0.142)	-0.104 (-0.21, 0.002)	-0.034 (-0.12, 0.052)	0.00056

© 2020 Ellis RP et al. *JAMA Network Open.*

CCS	Category label	9/15 Rate	α	β	γ	δ	$\gamma + \delta$	F: γ=δ=0
61	Sickle Cell Anemia	1.03 (0.98, 1.08)	0.53 (0.44, 0.61)	0.0059 (0.0046, 0.0071)	0.0956 (0.003, 0.188)	0.012 (-0.123, 0.148)	0.108 (-0.002, 0.218)	0.00002
62	Coagulation and Hemorrhagic Disorders	9 (8.84, 9.16)	0.54 (0.49, 0.6)	0.0054 (0.0046, 0.0062)	0.0413 (-0.02, 0.102)	-0.042 (-0.132, 0.048)	0.000 (-0.073, 0.073)	0.04715
63	Diseases of White Blood Cells	8.82 (8.67, 8.97)	0.46 (0.41, 0.52)	0.007 (0.0062, 0.0079)	0.0436 (-0.02, 0.108)	-0.019 (-0.113, 0.075)	0.024 (-0.052, 0.101)	0.03108
64	Other Hematologic Conditions	2.47 (2.39, 2.55)	0.33 (0.24, 0.41)	0.0081 (0.0068, 0.0094)	0.4744 (0.38, 0.568)	0.173 (0.035, 0.312)	0.648 (0.535, 0.76)	0
76	Meningitis (Except That Caused by Tuberculosis or Sexually Transmitted Disease)	0.46 (0.42, 0.5)	0.63 (0.55, 0.71)	0.0002 (-0.001, 0.0014)	0.0063 (-0.084, 0.097)	-0.064 (-0.197, 0.069)	-0.058 (-0.166, 0.05)	0.12872
77	Encephalitis (Except That Caused by Tuberculosis or Sexually Transmitted Disease)	0.39 (0.36, 0.42)	0.57 (0.5, 0.63)	0.0048 (0.0038, 0.0057)	-0.1631 (-0.235, -0.091)	-0.193 (-0.299, -0.087)	-0.356 (-0.442, -0.271)	0
78	Other CNS Infection and Poliomyelitis	0.2 (0.18, 0.22)	0.78 (0.7, 0.86)	0.0026 (0.0014, 0.0037)	0.3413 (0.254, 0.428)	-0.219 (-0.347, -0.091)	0.122 (0.018, 0.226)	0
79	Parkinson's Disease	1.16 (1.1, 1.22)	0.73 (0.67, 0.79)	0.0029 (0.002, 0.0038)	0.0125 (-0.055, 0.08)	-0.03 (-0.129, 0.069)	-0.018 (-0.098, 0.063)	0.53323
80	Multiple Sclerosis	5.63 (5.51, 5.75)	0.84 (0.79, 0.88)	0.0018 (0.001, 0.0025)	0.0082 (-0.045, 0.061)	-0.014 (-0.092, 0.065)	-0.005 (-0.069, 0.058)	0.78788
81	Other Hereditary and Degenerative Nervous System Conditions	10.19 (10.02, 10.36)	0.51 (0.45, 0.56)	0.0061 (0.0052, 0.007)	-0.0226 (-0.088, 0.043)	-0.060 (-0.157, 0.037)	-0.083 (-0.161, -0.004)	0.00069
82	Paralysis	3.84 (3.74, 3.94)	0.61 (0.56, 0.66)	0.0044 (0.0036, 0.0052)	0.0749 (0.018, 0.132)	-0.034 (-0.118, 0.05)	0.041 (-0.027, 0.109)	0.00001
83	Epilepsy; Convulsions	12.13 (11.95, 12.31)	0.76 (0.71, 0.81)	0.0033 (0.0026, 0.0041)	-0.0258 (-0.081, 0.029)	-0.044 (-0.125, 0.037)	-0.07 (-0.135, -0.004)	0.00039
84	Headache; Including Migraine	83.04 (82.57, 83.51)	0.68 (0.63, 0.74)	0.0043 (0.0035, 0.0052)	0.0519 (-0.01, 0.113)	-0.082 (-0.172, 0.009)	-0.03 (-0.103, 0.044)	0.00225
85	Coma; Stupor; and Brain Damage	1.35 (1.29, 1.41)	0.73 (0.24, 1.23)	0.01 (0.0027, 0.0174)	-0.6227 (-1.169, -0.076)	0.360 (-0.443, 1.164)	-0.262 (-0.914, 0.39)	0.0002
86	Cataract	27.79 (27.52, 28.06)	0.49 (0.42, 0.56)	0.006 (0.0049, 0.007)	0.0376 (-0.039, 0.114)	-0.091 (-0.204, 0.022)	-0.053 (-0.145, 0.038)	0.01419

© 2020 Ellis RP et al. *JAMA Network Open.*

CCS	Category label	9/15 Rate	α	β	γ	δ	$\gamma + \delta$	F: γ=δ=0
87	Retinal Detachments; Defects; Vascular Occlusion; and Retinopathy	21.8 (21.56, 22.04)	0.57 (0.52, 0.63)	0.0054 (0.0047, 0.0062)	-0.2205 (-0.278, -0.163)	-0.067 (-0.152, 0.018)	-0.288 (-0.357, -0.219)	0
88	Glaucoma	26.54 (26.27, 26.81)	0.83 (0.77, 0.89)	0.0024 (0.0015, 0.0033)	-0.3085 (-0.376, -0.241)	0.15 (0.05, 0.249)	-0.159 (-0.24, -0.078)	0
89	Blindness and Vision Defects	25.03 (24.77, 25.29)	0.64 (0.56, 0.72)	0.004 (0.0028, 0.0052)	0.5432 (0.453, 0.633)	-0.031 (-0.163, 0.101)	0.513 (0.406, 0.62)	0
90	Inflammation; Infection of Eye (Except that Caused by Tuberculosis or Sexually Transmitted disease)	51.05 (50.68, 51.42)	0.85 (0.78, 0.92)	0.0025 (0.0014, 0.0035)	0.046 (-0.032, 0.124)	-0.089 (-0.204, 0.026)	-0.043 (-0.136, 0.051)	0.01725
91	Other Eye Disorders	63.34 (62.93, 63.75)	0.68 (0.62, 0.73)	0.0037 (0.0029, 0.0045)	-0.0397 (-0.101, 0.021)	-0.047 (-0.137, 0.043)	-0.087 (-0.16, -0.014)	0.00004
92	Otitis Media and Related Conditions	69.23 (68.8, 69.66)	1.44 (1.32, 1.56)	-0.0002 (-0.002, 0.0016)	0.0609 (-0.072, 0.194)	0.012 (-0.185, 0.208)	0.073 (-0.087, 0.232)	0.08196
93	Conditions Associated with Dizziness or Vertigo	26.81 (26.54, 27.08)	0.65 (0.6, 0.7)	0.0039 (0.0031, 0.0046)	0.0024 (-0.055, 0.059)	-0.062 (-0.145, 0.022)	-0.059 (-0.127, 0.009)	0.00682
94	Other Ear and Sense Organ Disorders	62.19 (61.78, 62.6)	0.75 (0.69, 0.81)	0.0031 (0.0021, 0.004)	-0.0497 (-0.12, 0.02)	-0.047 (-0.15, 0.057)	-0.096 (-0.18, -0.013)	0.00004
95	Other Nervous System Disorders	116.29 (115.73, 116.85)	0.46 (0.4, 0.52)	0.0064 (0.0055, 0.0073)	0.0476 (-0.018, 0.113)	0.049 (-0.048, 0.145)	0.096 (0.018, 0.175)	0.00001
96	Heart Valve Disorders	21.57 (21.33, 21.81)	0.7 (0.63, 0.76)	0.0032 (0.0023, 0.0042)	-0.0138 (-0.086, 0.059)	-0.024 (-0.13, 0.082)	-0.038 (-0.124, 0.049)	0.23951
97	Peri-; Endo-; and Myocarditis; Cardiomyopathy (Except That Caused by Tuberculosis or Sexually Transmitted Disease)	6.59 (6.46, 6.72)	0.66 (0.6, 0.72)	0.0043 (0.0034, 0.0053)	0.07 (0, 0.14)	-0.115 (-0.218, -0.012)	-0.045 (-0.129, 0.039)	0.00017
98	Essential Hypertension	251.89 (251.07, 252.71)	0.66 (0.59, 0.72)	0.0046 (0.0036, 0.0056)	0.0512 (-0.02, 0.123)	-0.106 (-0.211, -0.001)	-0.055 (-0.14, 0.031)	0.00146
99	Hypertension with Complications and Secondary Hypertension	14.17 (13.97, 14.37)	0.81 (0.74, 0.88)	0.0011 (0, 0.0021)	-0.1405 (-0.218, -0.063)	0.182 (0.068, 0.296)	0.041 (-0.051, 0.134)	0
100	Acute Myocardial Infarction	1.97 (1.9, 2.04)	0.65 (0.6, 0.7)	0.0048 (0.0041, 0.0056)	0.0076 (-0.05, 0.065)	-0.005 (-0.089, 0.079)	0.003 (-0.066, 0.071)	0.88353
101	Coronary Atherosclerosis and Other Heart Disease	27.71 (27.44, 27.98)	0.9 (0.83, 0.96)	0.0005 (-0.0005, 0.0016)	0.0179 (-0.058, 0.094)	-0.002 (-0.114, 0.109)	0.016 (-0.075, 0.106)	0.58829

© 2020 Ellis RP et al. *JAMA Network Open.*

CCS	Category label	9/15 Rate	α	β	γ	δ	$\gamma + \delta$	F: γ=δ=0
102	Nonspecific Chest Pain	41.07 (40.74, 41.4)	0.94 (0.88, 0.99)	0.0006 (-0.0002, 0.0014)	0.0258 (-0.033, 0.085)	-0.007 (-0.094, 0.08)	0.019 (-0.052, 0.089)	0.19922
103	Pulmonary Heart Disease	5.02 (4.9, 5.14)	0.51 (0.46, 0.56)	0.0064 (0.0057, 0.0072)	0.0375 (-0.018, 0.093)	-0.131 (-0.212, -0.05)	-0.093 (-0.159, -0.028)	0
104	Other and Ill-Defined Heart Disease	5.26 (5.14, 5.38)	0.51 (0.42, 0.59)	0.0058 (0.0045, 0.007)	-0.0863 (-0.179, 0.006)	-0.071 (-0.207, 0.065)	-0.157 (-0.268, -0.047)	0
105	Conduction Disorders	5.21 (5.09, 5.33)	0.51 (0.45, 0.58)	0.0063 (0.0053, 0.0072)	0.0626 (-0.007, 0.132)	-0.085 (-0.188, 0.017)	-0.022 (-0.106, 0.061)	0.00244
106	Cardiac Dysrhythmias	44.56 (44.21, 44.91)	0.67 (0.62, 0.73)	0.0044 (0.0036, 0.0052)	0.0114 (-0.048, 0.071)	-0.023 (-0.11, 0.064)	-0.012 (-0.083, 0.059)	0.61037
107	Cardiac Arrest and Ventricular Fibrillation	0.57 (0.53, 0.61)	0.64 (0.59, 0.69)	0.0042 (0.0034, 0.0049)	-0.023 (-0.078, 0.032)	-0.129 (-0.21, -0.048)	-0.152 (-0.217, -0.086)	0
108	Congestive Heart Failure; Nonhypertensive	6.08 (5.95, 6.21)	0.67 (0.6, 0.74)	0.0041 (0.003, 0.0052)	0.0437 (-0.035, 0.123)	0.001 (-0.115, 0.117)	0.045 (-0.049, 0.139)	0.04204
109	Acute Cerebrovascular Disease	4.46 (4.35, 4.57)	0.63 (0.58, 0.69)	0.0043 (0.0035, 0.0052)	-0.1153 (-0.178, -0.053)	-0.03 (-0.121, 0.062)	-0.145 (-0.219, -0.071)	0
110	Occlusion or Stenosis of Precerebral Arteries	2.19 (2.11, 2.27)	0.52 (0.44, 0.59)	0.0059 (0.0048, 0.007)	-0.0144 (-0.096, 0.067)	-0.100 (-0.22, 0.02)	-0.115 (-0.212, -0.017)	0.00019
111	Other and Ill-Defined Cerebrovascular Disease	1.12 (1.06, 1.18)	0.62 (0.54, 0.7)	0.0043 (0.0031, 0.0055)	0.5614 (0.474, 0.649)	-0.249 (-0.378, -0.12)	0.313 (0.208, 0.417)	0
112	Transient Cerebral Ischemia	1.87 (1.8, 1.94)	0.78 (0.71, 0.84)	0.0021 (0.0011, 0.0031)	0.0533 (-0.02, 0.126)	-0.081 (-0.188, 0.027)	-0.028 (-0.115, 0.06)	0.01122
113	Late Effects of Cerebrovascular Disease	2.22 (2.14, 2.3)	0.77 (0.72, 0.83)	0.0032 (0.0024, 0.004)	-0.2658 (-0.326, -0.205)	-0.118 (-0.207, -0.029)	-0.383 (-0.456, -0.311)	0
114	Peripheral and Visceral Atherosclerosis	5.64 (5.52, 5.76)	0.62 (0.55, 0.68)	0.0044 (0.0034, 0.0054)	-0.0573 (-0.133, 0.019)	-0.113 (-0.225, -0.001)	-0.171 (-0.261, -0.08)	0
115	Aortic; Peripheral; and Visceral Artery Aneurysms	2.18 (2.1, 2.26)	0.4 (0.33, 0.47)	0.0079 (0.0069, 0.0089)	0.057 (-0.019, 0.133)	0.064 (-0.048, 0.175)	0.121 (0.03, 0.211)	0
116	Aortic and Peripheral Arterial Embolism or Thrombosis	0.36 (0.33, 0.39)	0.86 (0.79, 0.93)	0.0012 (0.0002, 0.0023)	0.0839 (0.007, 0.161)	-0.065 (-0.178, 0.048)	0.019 (-0.073, 0.11)	0.00053
117	Other Circulatory Disease	39.1 (38.77, 39.43)	0.51 (0.44, 0.58)	0.0068 (0.0058, 0.0078)	-0.2884 (-0.364, -0.213)	0.093 (-0.018, 0.204)	-0.195 (-0.286, -0.105)	0
118	Phlebitis; Thrombophlebitis and Thromboembolism	7.52 (7.38, 7.66)	0.67 (0.63, 0.72)	0.0039 (0.0032, 0.0046)	-0.0625 (-0.113, -0.012)	-0.103 (-0.178, -0.028)	-0.166 (-0.226, -0.105)	0
CCS	Category label	9/15 Rate	α	β	γ	δ	$\gamma + \delta$	F: γ=δ=0

© 2020 Ellis RP et al. *JAMA Network Open.*

119	Varicose Veins of Lower Extremity	7.89 (7.74, 8.04)	0.61 (0.55, 0.67)	0.0028 (0.0019, 0.0037)	-0.0608 (-0.129, 0.007)	-0.001 (-0.101, 0.098)	-0.062 (-0.143, 0.019)	0.00039
120	Hemorrhoids	18.13 (17.91, 18.35)	0.62 (0.56, 0.69)	0.0043 (0.0033, 0.0052)	-0.0212 (-0.093, 0.051)	-0.003 (-0.109, 0.102)	-0.024 (-0.11, 0.061)	0.35853
121	Other Diseases of Veins and Lymphatics	8.02 (7.87, 8.17)	0.44 (0.38, 0.51)	0.0056 (0.0046, 0.0066)	0.0722 (0, 0.144)	-0.072 (-0.177, 0.034)	0.001 (-0.085, 0.087)	0.0016
122	Pneumonia (Except That Caused by Tuberculosis or Sexually Transmitted Disease)	9.81 (9.65, 9.97)	1.65 (1.5, 1.79)	-0.0014 (-0.0036, 0.0008)	0.2303 (0.066, 0.394)	-0.213 (-0.454, 0.028)	0.017 (-0.178, 0.213)	0.00001
123	Influenza	1.9 (1.83, 1.97)	12.67 (2.4, 22.95)	0.1218 (-0.0312, 0.2748)	-6.1925 (-17.587, 5.202)	5.81 (-10.95, 22.57)	-0.383 (-13.98, 13.21)	0.13742
124	Acute and Chronic Tonsillitis	13.18 (12.99, 13.37)	1.17 (1.07, 1.26)	-0.0014 (-0.0028, 0)	0.1211 (0.016, 0.226)	-0.02 (-0.174, 0.135)	0.102 (-0.024, 0.227)	0.00002
125	Acute Bronchitis	34.11 (33.81, 34.41)	2.13 (1.94, 2.33)	-0.003 (-0.006, 0)	-0.0091 (-0.23, 0.211)	-0.048 (-0.372, 0.277)	-0.057 (-0.32, 0.206)	0.7267
126	Other Upper Respiratory Infections	277.73 (276.87, 278.59)	1.33 (1.22, 1.45)	0.0013 (-0.0004, 0.003)	0.0009 (-0.123, 0.125)	0.005 (-0.178, 0.188)	0.006 (-0.142, 0.154)	0.98897
127	Chronic Obstructive Pulmonary Disease and Bronchiectasis	24.8 (24.54, 25.06)	1.11 (1.01, 1.22)	0.0037 (0.0021, 0.0052)	0.0169 (-0.099, 0.132)	-0.219 (-0.389, -0.049)	-0.202 (-0.339, -0.064)	0
128	Asthma	68.56 (68.13, 68.99)	0.81 (0.75, 0.87)	0.0034 (0.0025, 0.0043)	-0.0111 (-0.079, 0.057)	-0.063 (-0.163, 0.037)	-0.074 (-0.155, 0.007)	0.0047
129	Aspiration Pneumonitis; Food/Vomitus	0.31 (0.28, 0.34)	0.55 (0.47, 0.64)	0.0048 (0.0035, 0.0061)	0.1879 (0.092, 0.284)	-0.163 (-0.304, -0.022)	0.024 (-0.09, 0.139)	0
130	Pleurisy; Pneumothorax; Pulmonary Collapse	2.78 (2.69, 2.87)	0.86 (0.8, 0.92)	0.0031 (0.0022, 0.004)	0.0144 (-0.053, 0.082)	-0.081 (-0.18, 0.018)	-0.067 (-0.147, 0.013)	0.00617
131	Respiratory Failure; Insufficiency; Arrest (Adult)	2.85 (2.76, 2.94)	0.78 (0.71, 0.84)	0.0041 (0.0032, 0.0051)	-0.0157 (-0.085, 0.054)	-0.099 (-0.201, 0.004)	-0.114 (-0.198, -0.031)	0.00001
132	Lung Disease Due to External Agents	0.91 (0.86, 0.96)	0.42 (0.11, 0.72)	0.0092 (0.0046, 0.0138)	-0.5903 (-0.933, -0.248)	-0.340 (-0.843, 0.164)	-0.930 (-1.338, -0.521)	0
133	Other Lower Respiratory Disease	104.87 (104.34, 105.4)	0.88 (0.75, 1.01)	0.0066 (0.0047, 0.0085)	0.0409 (-0.101, 0.183)	-0.054 (-0.263, 0.155)	-0.013 (-0.183, 0.156)	0.52412
134	Other Upper Respiratory Disease	183.72 (183.02, 184.42)	0.7 (0.64, 0.76)	0.0031 (0.0022, 0.004)	0.0116 (-0.054, 0.077)	-0.064 (-0.16, 0.033)	-0.052 (-0.13, 0.026)	0.03558

© 2020 Ellis RP et al. *JAMA Network Open.*

CCS	Category label	9/15 Rate	α	β	γ	δ	$\gamma + \delta$	F: γ=δ=0
135	Intestinal Infection	6.69 (6.56, 6.82)	1.14 (1.01, 1.27)	0.0026 (0.0007, 0.0045)	0.107 (-0.035, 0.249)	0.128 (-0.08, 0.337)	0.235 (0.066, 0.404)	0
136	Disorders of Teeth and Jaw	12.25 (12.07, 12.43)	0.74 (0.68, 0.81)	0.0032 (0.0023, 0.0042)	0.0629 (-0.007, 0.133)	-0.085 (-0.188, 0.019)	-0.022 (-0.106, 0.062)	0.00266
137	Diseases of Mouth; Excluding Dental	8.28 (8.13, 8.43)	0.61 (0.55, 0.67)	0.005 (0.0041, 0.0059)	0.0265 (-0.041, 0.094)	-0.040 (-0.139, 0.059)	-0.013 (-0.094, 0.067)	0.26036
138	Esophageal Disorders	74.46 (74.01, 74.91)	0.58 (0.51, 0.65)	0.0059 (0.0049, 0.0069)	0.0265 (-0.049, 0.102)	-0.096 (-0.207, 0.014)	-0.07 (-0.159, 0.02)	0.00569
139	Gastroduodenal Ulcer (Except Hemorrhage)	2.42 (2.34, 2.5)	0.7 (0.64, 0.76)	0.0032 (0.0022, 0.0041)	0.0553 (-0.015, 0.125)	-0.054 (-0.157, 0.049)	0.001 (-0.082, 0.084)	0.0164
140	Gastritis and Duodenitis	13.13 (12.94, 13.32)	0.81 (0.75, 0.86)	0.0022 (0.0014, 0.003)	0.0115 (-0.049, 0.072)	-0.061 (-0.149, 0.028)	-0.049 (-0.121, 0.023)	0.02963
141	Other Disorders of Stomach and Duodenum	9.22 (9.06, 9.38)	0.56 (0.5, 0.62)	0.0053 (0.0044, 0.0062)	-0.1972 (-0.267, -0.128)	-0.143 (-0.245, -0.04)	-0.340 (-0.423, -0.257)	0
142	Appendicitis and Other Appendiceal Conditions	1.53 (1.47, 1.59)	0.89 (0.85, 0.94)	-0.0004 (-0.001, 0.0003)	0.006 (-0.044, 0.056)	0.014 (-0.06, 0.088)	0.02 (-0.04, 0.08)	0.43934
143	Abdominal Hernia	13.06 (12.87, 13.25)	0.72 (0.67, 0.78)	0.003 (0.0022, 0.0039)	0.0522 (-0.012, 0.116)	-0.011 (-0.105, 0.084)	0.041 (-0.035, 0.118)	0.00397
144	Regional Enteritis and Ulcerative Colitis	10.98 (10.81, 11.15)	0.68 (0.63, 0.73)	0.0041 (0.0034, 0.0048)	0.0332 (-0.021, 0.088)	0.028 (-0.052, 0.108)	0.061 (-0.004, 0.126)	0.0008
145	Intestinal Obstruction Without Hernia	1.74 (1.67, 1.81)	0.8 (0.75, 0.84)	0.0031 (0.0024, 0.0037)	-0.0576 (-0.106, -0.009)	-0.096 (-0.167, -0.026)	-0.154 (-0.212, -0.097)	0
146	Diverticulosis and Diverticulitis	13.82 (13.63, 14.01)	0.61 (0.55, 0.68)	0.0039 (0.0029, 0.0049)	0.0251 (-0.048, 0.098)	-0.027 (-0.134, 0.081)	-0.001 (-0.089, 0.086)	0.43791
147	Anal and Rectal Conditions	7.67 (7.53, 7.81)	0.67 (0.61, 0.74)	0.0037 (0.0028, 0.0046)	0.1374 (0.069, 0.206)	-0.033 (-0.134, 0.068)	0.104 (0.022, 0.186)	0
148	Peritonitis and Intestinal Abscess	0.72 (0.68, 0.76)	0.54 (0.49, 0.59)	0.0047 (0.0039, 0.0054)	-0.0547 (-0.11, 0.001)	-0.073 (-0.154, 0.009)	-0.127 (-0.194, -0.061)	0
149	Biliary Tract Disease	6.71 (6.58, 6.84)	0.88 (0.83, 0.93)	0.0005 (-0.0002, 0.0012)	0.0156 (-0.038, 0.069)	-0.002 (-0.081, 0.077)	0.014 (-0.05, 0.077)	0.44755

© 2020 Ellis RP et al. *JAMA Network Open.*

CCS	Category label	9/15 Rate	α	β	γ	δ	$\gamma + \delta$	F: γ=δ=0
151	Other Liver Diseases	17.46 (17.24, 17.68)	0.5 (0.44, 0.57)	0.0063 (0.0053, 0.0073)	-0.1312 (-0.205, -0.057)	0.002 (-0.107, 0.111)	-0.13 (-0.218, -0.041)	0
152	Pancreatic Disorders (not Diabetes)	2.69 (2.6, 2.78)	0.65 (0.6, 0.7)	0.0041 (0.0033, 0.0048)	0.0338 (-0.021, 0.089)	-0.064 (-0.145, 0.017)	-0.03 (-0.096, 0.036)	0.01414
153	Gastrointestinal Hemorrhage	12.93 (12.74, 13.12)	0.94 (0.88, 1)	0.0004 (-0.0005, 0.0014)	-0.0063 (-0.075, 0.062)	-0.031 (-0.132, 0.07)	-0.038 (-0.119, 0.044)	0.23932
154	Noninfectious Gastroenteritis	11.71 (11.53, 11.89)	1.16 (1.03, 1.29)	0.002 (0.0001, 0.004)	-0.0481 (-0.195, 0.099)	0.001 (-0.214, 0.217)	-0.047 (-0.222, 0.128)	0.33342
155	Other Gastrointestinal Disorders	81.94 (81.47, 82.41)	0.57 (0.51, 0.63)	0.0055 (0.0046, 0.0063)	0.0665 (0, 0.133)	-0.079 (-0.177, 0.018)	-0.013 (-0.092, 0.066)	0.00106
156	Nephritis; Nephrosis; Renal Sclerosis	1.85 (1.78, 1.92)	0.57 (0.5, 0.65)	0.0059 (0.0048, 0.007)	-0.2463 (-0.328, -0.164)	-0.073 (-0.193, 0.047)	-0.319 (-0.417, -0.222)	0
157	Acute and Unspecified Renal Failure	3.74 (3.64, 3.84)	0.51 (0.45, 0.57)	0.0057 (0.0048, 0.0066)	0.0204 (-0.048, 0.089)	-0.09 (-0.191, 0.01)	-0.07 (-0.152, 0.012)	0.00344
158	Chronic Kidney Disease	16.06 (15.85, 16.27)	0.6 (0.54, 0.65)	0.0044 (0.0036, 0.0053)	0.0638 (0, 0.128)	-0.089 (-0.183, 0.005)	-0.025 (-0.102, 0.051)	0.00058
159	Urinary Tract Infections	44.94 (44.59, 45.29)	0.76 (0.72, 0.8)	0.002 (0.0013, 0.0026)	0.0039 (-0.042, 0.05)	-0.116 (-0.183, -0.048)	-0.112 (-0.167, -0.057)	0
160	Calculus of Urinary Tract	14.7 (14.5, 14.9)	0.67 (0.62, 0.71)	0.0038 (0.0031, 0.0045)	-0.0011 (-0.051, 0.048)	-0.085 (-0.158, -0.012)	-0.086 (-0.145, -0.027)	0
161	Other Diseases of Kidney and Ureters	9.04 (8.88, 9.2)	0.54 (0.48, 0.6)	0.0056 (0.0047, 0.0066)	0.0551 (-0.013, 0.124)	0.028 (-0.073, 0.129)	0.083 (0.001, 0.165)	0.00009
162	Other Diseases of Bladder and Urethra	5.33 (5.21, 5.45)	0.67 (0.6, 0.74)	0.0036 (0.0026, 0.0046)	0.0276 (-0.05, 0.105)	-0.064 (-0.178, 0.05)	-0.036 (-0.128, 0.056)	0.12376
163	Genitourinary Symptoms and Ill-Defined Conditions	64.4 (63.98, 64.82)	0.53 (0.47, 0.58)	0.0058 (0.0049, 0.0066)	0.0414 (-0.021, 0.104)	-0.063 (-0.155, 0.03)	-0.021 (-0.096, 0.054)	0.02526
164	Hyperplasia of Prostate	13.05 (12.86, 13.24)	0.66 (0.59, 0.73)	0.0044 (0.0033, 0.0054)	0.0161 (-0.062, 0.094)	-0.076 (-0.19, 0.039)	-0.06 (-0.152, 0.033)	0.03899
165	Inflammatory Conditions of Male Genital Organs	5.14 (5.02, 5.26)	0.98 (0.93, 1.03)	0.0001 (-0.0007, 0.0009)	0.0189 (-0.041, 0.079)	-0.104 (-0.193, -0.015)	-0.085 (-0.157, -0.013)	0.00006
166	Other Male Genital Disorders	19.51 (19.28, 19.74)	0.54 (0.48, 0.6)	0.0063 (0.0054, 0.0072)	0.0308 (-0.038, 0.1)	-0.116 (-0.217, -0.015)	-0.085 (-0.167, -0.003)	0.00019

© 2020 Ellis RP et al. *JAMA Network Open.*

CCS	Category label	9/15 Rate	α	β	γ	δ	$\gamma + \delta$	F: γ=δ=0
167	Nonmalignant Breast Conditions	21.31 (21.07, 21.55)	0.84 (0.78, 0.91)	0.0019 (0.0009, 0.0029)	-0.0697 (-0.143, 0.004)	-0.051 (-0.159, 0.057)	-0.121 (-0.208, -0.033)	0
168	Inflammatory Diseases of Female Pelvic Organs	19.7 (19.47, 19.93)	0.84 (0.79, 0.9)	0.0017 (0.0009, 0.0025)	-0.0172 (-0.077, 0.043)	-0.068 (-0.157, 0.021)	-0.085 (-0.157, -0.013)	0.00016
169	Endometriosis	3.14 (3.05, 3.23)	0.7 (0.64, 0.76)	0.0032 (0.0022, 0.0041)	0.0481 (-0.023, 0.119)	-0.046 (-0.151, 0.058)	0.002 (-0.083, 0.087)	0.04787
170	Prolapse of Female Genital Organs	3.11 (3.02, 3.2)	0.76 (0.69, 0.83)	0.0016 (0.0006, 0.0026)	0.1136 (0.038, 0.189)	0.006 (-0.105, 0.118)	0.12 (0.03, 0.21)	0
171	Menstrual Disorders	42.83 (42.49, 43.17)	0.8 (0.74, 0.86)	0.0023 (0.0015, 0.0032)	-0.0832 (-0.148, -0.018)	-0.055 (-0.15, 0.041)	-0.138 (-0.215, -0.06)	0
172	Ovarian Cyst	7.68 (7.54, 7.82)	0.65 (0.59, 0.71)	0.0043 (0.0034, 0.0051)	-0.0524 (-0.115, 0.01)	-0.168 (-0.26, -0.076)	-0.22 (-0.295, -0.146)	0
173	Menopausal Disorders	24.87 (24.61, 25.13)	0.57 (0.5, 0.64)	0.0043 (0.0033, 0.0053)	0.0269 (-0.048, 0.102)	-0.151 (-0.262, -0.041)	-0.124 (-0.214, -0.035)	0
174	Female Infertility	4.92 (4.8, 5.04)	0.88 (0.84, 0.93)	0.0005 (-0.0001, 0.0012)	0.0454 (-0.006, 0.097)	0 (-0.076, 0.076)	0.045 (-0.016, 0.107)	0.00055
175	Other Female Genital Disorders	39.82 (39.49, 40.15)	0.7 (0.65, 0.76)	0.0031 (0.0022, 0.0039)	-0.0841 (-0.147, -0.021)	-0.034 (-0.127, 0.059)	-0.118 (-0.193, -0.042)	0
176	Contraceptive and Procreative Management	48.37 (48.01, 48.73)	0.53 (0.47, 0.58)	0.0068 (0.006, 0.0076)	-0.0572 (-0.12, 0.005)	0.005 (-0.087, 0.097)	-0.053 (-0.127, 0.022)	0.00054
177	Spontaneous Abortion	1.16 (1.1, 1.22)	0.96 (0.9, 1.01)	0.001 (0.0002, 0.0019)	0.0639 (-0.001, 0.129)	-0.003 (-0.098, 0.092)	0.061 (-0.016, 0.138)	0.00014
178	Induced Abortion	0.48 (0.44, 0.52)	1.09 (1.02, 1.15)	0.0006 (-0.0004, 0.0015)	-0.4602 (-0.532, -	-0.084 (-0.19, 0.021)	-0.544 (-0.63, -0.459)	0
179	Postabortion Complications	0.03 (0.02, 0.04)	1.26 (1.03, 1.49)	-0.0008 (-0.0043, 0.0026)	-0.0296 (-0.286, 0.227)	-0.460 (-0.837, -0.082)	-0.489 (-0.795, -0.183)	0
180	Ectopic Pregnancy	0.33 (0.3, 0.36)	0.89 (0.82, 0.95)	0.0016 (0.0006, 0.0025)	-0.0087 (-0.083, 0.066)	-0.240 (-0.349, -0.13)	-0.248 (-0.337, -0.16)	0
181	Other Complications of Pregnancy	11.45 (11.27, 11.63)	0.61 (0.57, 0.66)	0.0052 (0.0045, 0.0059)	0.6659 (0.613,	0.032 (-0.046, 0.109)	0.697 (0.634, 0.761)	0

© 2020 Ellis RP et al. *JAMA Network Open.*

CCS	Category label	9/15 Rate	α	β	γ	δ	$\gamma + \delta$	F: γ=δ=0
182	Hemorrhage During Pregnancy; Abruptio Placenta; Placenta Previa	2.92<br>(2.83, 3.01)	0.89<br>(0.85, 0.92)	0.002<br>(0.0014, 0.0025)	-0.037<br>(-0.076, 0.002)	-0.027<br>(-0.085, 0.031)	-0.064<br>(-0.111, -0.017)	0
183	Hypertension Complicating Pregnancy; Childbirth and the Puerperium	2.22<br>(2.14, 2.3)	0.63<br>(0.58, 0.68)	0.0041<br>(0.0033, 0.0048)	0.0026<br>(-0.056, 0.061)	0.059<br>(-0.027, 0.145)	0.062<br>(-0.008, 0.132)	0.00661
184	Early or Threatened Labor	2.57<br>(2.49, 2.65)	0.91<br>(0.85, 0.96)	0.0001<br>(-0.0007, 0.0009)	-0.0876<br>(-0.149, -0.026)	-0.051<br>(-0.142, 0.041)	-0.138<br>(-0.212, -0.064)	0
185	Prolonged Pregnancy	1.04<br>(0.99, 1.09)	0.5<br>(0.44, 0.56)	0.0046<br>(0.0038, 0.0055)	0.005<br>(-0.058, 0.068)	0.022<br>(-0.071, 0.115)	0.027<br>(-0.048, 0.102)	0.41602
186	Diabetes or Abnormal Glucose Tolerance Complicating Pregnancy; Childbirth; or the Puerperium	2.45<br>(2.37, 2.53)	0.65<br>(0.61, 0.69)	0.0033<br>(0.0026, 0.0039)	0.067<br>(0.02, 0.114)	0.100<br>(0.031, 0.169)	0.167<br>(0.111, 0.223)	0
187	Malposition; Malpresentation	0.81<br>(0.76, 0.86)	0.74<br>(0.69, 0.8)	0.0029<br>(0.0021, 0.0037)	-0.0408<br>(-0.101, 0.02)	-0.032<br>(-0.121, 0.057)	-0.073<br>(-0.145, -0.001)	0.00024
188	Fetopelvic Disproportion; Obstruction	0.55<br>(0.51, 0.59)	1.04<br>(0.96, 1.11)	-0.0019<br>(-0.0031, -0.0008)	-0.0078<br>(-0.094, 0.078)	-0.163<br>(-0.289, -0.037)	-0.171<br>(-0.273, -0.068)	0
189	Previous C-Section	2.64<br>(2.56, 2.72)	0.65<br>(0.6, 0.7)	0.0029<br>(0.0022, 0.0036)	0.0057<br>(-0.045, 0.057)	-0.13<br>(-0.205, -0.055)	-0.124<br>(-0.185, -0.063)	0
190	Fetal Distress and Abnormal Forces of Labor	1.19<br>(1.13, 1.25)	0.77<br>(0.72, 0.82)	0.0015<br>(0.0007, 0.0022)	-0.0412<br>(-0.097, 0.014)	0.086<br>(0.005, 0.168)	0.045<br>(-0.021, 0.112)	0.00078
191	Polyhydramnios and Other Problems of Amniotic Cavity	1.35<br>(1.29, 1.41)	0.52<br>(0.47, 0.57)	0.0048<br>(0.004, 0.0055)	0.0056<br>(-0.05, 0.061)	0.090<br>(0.008, 0.171)	0.095<br>(0.029, 0.161)	0
192	Umbilical Cord Complication	0.81<br>(0.76, 0.86)	0.36<br>(0.29, 0.44)	0.0067<br>(0.0055, 0.0079)	-0.0535<br>(-0.14, 0.033)	0.074<br>(-0.053, 0.201)	0.021<br>(-0.082, 0.124)	0.0491
193	OB-Related Trauma to Perineum and Vulva	1.86<br>(1.79, 1.93)	0.47<br>(0.41, 0.52)	0.0053<br>(0.0045, 0.0062)	0.0489<br>(-0.014, 0.111)	0.024<br>(-0.068, 0.116)	0.073<br>(-0.001, 0.148)	0.00016
194	Forceps Delivery	0.19<br>(0.17, 0.21)	0.85<br>(0.76, 0.94)	0.0015<br>(0.0002, 0.0028)	-0.1067<br>(-0.206, -0.007)	-0.125<br>(-0.272, 0.021)	-0.232<br>(-0.351, -0.113)	0
195	Other Complications of Birth; Puerperium Affecting Management of Mother	14.64<br>(14.44, 14.84)	0.76<br>(0.72, 0.79)	0.0026<br>(0.0021, 0.0031)	-0.5877<br>(-0.626, -0.55)	-0.036<br>(-0.092, 0.02)	-0.624<br>(-0.669, -0.578)	0

© 2020 Ellis RP et al. *JAMA Network Open.*

CCS	Category label	9/15 Rate	α	β	γ	δ	$\gamma+\delta$	F: γ=δ=0
196	Other Pregnancy and Delivery Including Normal	23.04 (22.79, 23.29)	0.74 (0.7, 0.78)	0.0032 (0.0026, 0.0037)	0.0269 (-0.015, 0.069)	-0.103 (-0.164, -0.041)	-0.076 (-0.126, -0.026)	0
197	Skin and Subcutaneous Tissue Infections	44.03 (43.68, 44.38)	0.69 (0.65, 0.73)	0.0012 (0.0006, 0.0018)	-0.0093 (-0.053, 0.034)	-0.005 (-0.07, 0.059)	-0.015 (-0.067, 0.038)	0.46975
198	Other Inflammatory Condition of Skin	44.03 (43.68, 44.38)	0.59 (0.52, 0.65)	0.0048 (0.0038, 0.0058)	0.0973 (0.025, 0.169)	0.064 (-0.042, 0.17)	0.162 (0.076, 0.247)	0
199	Chronic Ulcer of Skin	5.3 (5.18, 5.42)	0.7 (0.65, 0.75)	0.003 (0.0023, 0.0038)	-0.0649 (-0.121, -0.008)	-0.025 (-0.108, 0.058)	-0.09 (-0.158, -0.023)	0
200	Other Skin Disorders	177.62 (176.93, 178.31)	0.66 (0.6, 0.72)	0.0036 (0.0027, 0.0045)	0.0627 (-0.004, 0.13)	-0.016 (-0.114, 0.083)	0.047 (-0.033, 0.127)	0.00087
201	Infective Arthritis and Osteomyelitis (Except That Caused by Tuberculosis or Sexually Transmitted Disease)	1.81 (1.74, 1.88)	0.7 (0.65, 0.75)	0.0031 (0.0023, 0.0038)	0.0336 (-0.023, 0.09)	-0.067 (-0.149, 0.016)	-0.033 (-0.1, 0.034)	0.0121
202	Rheumatoid Arthritis and Related Disease	16.38 (16.17, 16.59)	0.75 (0.7, 0.8)	0.0028 (0.0021, 0.0036)	0.0256 (-0.031, 0.082)	-0.090 (-0.173, -0.006)	-0.064 (-0.132, 0.004)	0.00052
203	Osteoarthritis	56.72 (56.33, 57.11)	0.63 (0.57, 0.69)	0.0043 (0.0034, 0.0052)	0.0475 (-0.02, 0.115)	-0.043 (-0.143, 0.056)	0.004 (-0.076, 0.085)	0.03831
204	Other Non-Traumatic Joint Disorders	192.93 (192.21, 193.65)	0.54 (0.49, 0.59)	0.0054 (0.0047, 0.0061)	-0.0784 (-0.131, -0.026)	-0.043 (-0.12, 0.035)	-0.121 (-0.184, -0.058)	0
205	Spondylosis; Intervertebral Disc Disorders; Other Back Problems	310.69 (309.79, 311.59)	0.69 (0.64, 0.75)	0.0035 (0.0027, 0.0042)	0.0375 (-0.02, 0.095)	-0.065 (-0.15, 0.02)	-0.027 (-0.096, 0.042)	0.01519
206	Osteoporosis	6.18 (6.05, 6.31)	0.7 (0.62, 0.78)	0.0028 (0.0016, 0.004)	0.0119 (-0.078, 0.102)	-0.032 (-0.164, 0.101)	-0.02 (-0.127, 0.087)	0.67293
207	Pathological Fracture	2.17 (2.09, 2.25)	0.56 (0.51, 0.62)	0.0037 (0.0028, 0.0045)	-0.6488 (-0.714, -0.584)	-0.105 (-0.201, -0.009)	-0.754 (-0.832, -0.676)	0
208	Acquired Foot Deformities	16.6 (16.39, 16.81)	0.62 (0.56, 0.68)	0.0035 (0.0026, 0.0044)	0.0003 (-0.067, 0.067)	-0.045 (-0.143, 0.054)	-0.044 (-0.124, 0.036)	0.12512
209	Other Acquired Deformities	12.21 (12.03, 12.39)	0.55 (0.47, 0.64)	0.0047 (0.0035, 0.006)	0.5162 (0.422, 0.61)	0.075 (-0.064, 0.213)	0.591 (0.478, 0.703)	0

© 2020 Ellis RP et al. *JAMA Network Open.*

CCS	Category label	9/15 Rate	α	β	γ	δ	$\gamma + \delta$	F: γ=δ=0
210	Systemic Lupus Erythematosus and Connective Tissue Disorders	8.23<br>(8.08, 8.38)	0.62<br>(0.57, 0.67)	0.0047<br>(0.0039, 0.0054)	0.0879<br>(0.029, 0.147)	-0.043<br>(-0.131, 0.044)	0.044<br>(-0.026, 0.115)	0
211	Other Connective Tissue Disease	270.78<br>(269.93, 271.63)	0.57<br>(0.51, 0.63)	0.0048<br>(0.0039, 0.0056)	-0.0358<br>(-0.1, 0.028)	-0.09<br>(-0.184, 0.005)	-0.126<br>(-0.202, -0.049)	0
212	Other Bone Disease and Musculoskeletal Deformities	144.45<br>(143.83, 145.07)	0.8<br>(0.73, 0.86)	0.0024<br>(0.0014, 0.0033)	0.0045<br>(-0.067, 0.076)	-0.023<br>(-0.127, 0.082)	-0.018<br>(-0.103, 0.067)	0.69529
213	Cardiac and Circulatory Congenital Anomalies	5.5<br>(5.38, 5.62)	0.69<br>(0.63, 0.74)	0.0036<br>(0.0027, 0.0044)	0.0399<br>(-0.022, 0.102)	0.019<br>(-0.072, 0.111)	0.059<br>(-0.015, 0.133)	0.00239
214	Digestive Congenital Anomalies	2.01<br>(1.94, 2.08)	0.48<br>(0.41, 0.54)	0.0061<br>(0.0052, 0.0071)	-0.0226<br>(-0.094, 0.048)	0.015<br>(-0.089, 0.12)	-0.008<br>(-0.092, 0.077)	0.49309
215	Genitourinary Congenital Anomalies	4.1<br>(3.99, 4.21)	0.55<br>(0.49, 0.61)	0.0047<br>(0.0038, 0.0056)	-0.0046<br>(-0.071, 0.062)	-0.159<br>(-0.257, -0.061)	-0.164<br>(-0.243, -0.084)	0
216	Nervous System Congenital Anomalies	1.42<br>(1.36, 1.48)	0.61<br>(0.55, 0.67)	0.0048<br>(0.0039, 0.0057)	0.272<br>(0.204, 0.34)	-0.039<br>(-0.14, 0.061)	0.232<br>(0.151, 0.314)	0
217	Other Congenital Anomalies	19.71<br>(19.48, 19.94)	0.58<br>(0.52, 0.64)	0.0046<br>(0.0037, 0.0055)	-0.1161<br>(-0.183, -0.049)	-0.021<br>(-0.12, 0.077)	-0.138<br>(-0.217, -0.058)	0
218	Liveborn	7.4<br>(7.26, 7.54)	0.95<br>(0.91, 1)	-0.0013<br>(-0.002, -0.0006)	-0.0083<br>(-0.059, 0.042)	0.033<br>(-0.042, 0.107)	0.024<br>(-0.036, 0.085)	0.25127
219	Short Gestation; Low Birth Weight; and Fetal Growth Retardation	1.69<br>(1.62, 1.76)	0.9<br>(0.83, 0.97)	0.0018<br>(0.0007, 0.0029)	-0.0772<br>(-0.158, 0.004)	0.251<br>(0.131, 0.37)	0.173<br>(0.077, 0.27)	0
220	Intrauterine Hypoxia and Birth Asphyxia	0.18<br>(0.16, 0.2)	0.75<br>(0.65, 0.85)	0.0031<br>(0.0016, 0.0045)	-0.4536<br>(-0.561, -0.346)	0.112<br>(-0.047, 0.27)	-0.342<br>(-0.471, -0.214)	0
221	Respiratory Distress Syndrome	0.31<br>(0.28, 0.34)	0.77<br>(0.69, 0.85)	0.0014<br>(0.0002, 0.0025)	-0.0267<br>(-0.114, 0.06)	-0.06<br>(-0.188, 0.067)	-0.087<br>(-0.191, 0.017)	0.00772
222	Hemolytic Jaundice and Perinatal Jaundice	2.54<br>(2.46, 2.62)	0.74<br>(0.68, 0.81)	0.0019<br>(0.0009, 0.0029)	0.0839<br>(0.007, 0.161)	0.003<br>(-0.11, 0.116)	0.087<br>(-0.004, 0.179)	0.00001
223	Birth Trauma	0.19<br>(0.17, 0.21)	0.6<br>(0.48, 0.71)	0.0035<br>(0.0017, 0.0053)	0.1409<br>(0.01, 0.272)	0.251<br>(0.058, 0.444)	0.392<br>(0.235, 0.548)	0
224	Other Perinatal Conditions	8.8<br>(8.65, 8.95)	0.74<br>(0.7, 0.78)	0.0021<br>(0.0015, 0.0027)	0.0622<br>(0.015, 0.11)	0.029<br>(-0.041, 0.099)	0.092<br>(0.035, 0.148)	0

© 2020 Ellis RP et al. *JAMA Network Open.*

CCS	Category label	9/15 Rate	α	β	γ	δ	$\gamma + \delta$	F: γ=δ=0
225	Joint Disorders and Dislocations; Trauma-Related	37.86 (37.54, 38.18)	1.2 (1.14, 1.26)	-0.0035 (-0.0044, -0.0026)	-0.0946 (-0.159, -0.03)	0.043 (-0.051, 0.138)	-0.051 (-0.128, 0.025)	0
226	Fracture of Neck of Femur (hip)	0.46 (0.42, 0.5)	0.79 (0.72, 0.86)	0.0018 (0.0008, 0.0028)	0.1431 (0.067, 0.219)	0.07 (-0.041, 0.182)	0.213 (0.123, 0.304)	0
227	Spinal Cord Injury	0.37 (0.34, 0.4)	0.68 (0.62, 0.74)	0.0029 (0.0021, 0.0038)	-0.1959 (-0.262, -0.13)	-0.079 (-0.176, 0.019)	-0.274 (-0.353, -0.196)	0
228	Skull and Face Fractures	1.28 (1.22, 1.34)	0.91 (0.86, 0.97)	-0.0004 (-0.0013, 0.0005)	0.0222 (-0.043, 0.087)	0.085 (-0.011, 0.18)	0.107 (0.03, 0.184)	0.00001
229	Fracture of Upper Limb	16.64 (16.43, 16.85)	0.76 (0.72, 0.79)	0 (-0.0005, 0.0005)	0.0209 (-0.018, 0.06)	0.004 (-0.053, 0.061)	0.025 (-0.021, 0.071)	0.03158
230	Fracture of Lower Limb	13.87 (13.68, 14.06)	0.72 (0.68, 0.76)	0.001 (0.0004, 0.0015)	0.1165 (0.074, 0.159)	0.017 (-0.046, 0.08)	0.133 (0.082, 0.184)	0
231	Other Fractures	5.02 (4.9, 5.14)	0.64 (0.6, 0.68)	0.0031 (0.0026, 0.0037)	-0.1888 (-0.231, -0.146)	-0.075 (-0.138, -0.013)	-0.264 (-0.315, -0.213)	0
232	Sprains and Strains	99.18 (98.66, 99.7)	0.84 (0.79, 0.88)	0.0011 (0.0004, 0.0018)	-0.1542 (-0.205, -0.104)	-0.056 (-0.13, 0.019)	-0.210 (-0.271, -0.15)	0
233	Intracranial Injury	6.86 (6.72, 7)	0.51 (0.44, 0.58)	0.0055 (0.0045, 0.0066)	-0.0634 (-0.141, 0.014)	-0.107 (-0.222, 0.007)	-0.171 (-0.264, -0.078)	0
234	Crushing Injury or Internal Injury	1.56 (1.49, 1.63)	0.75 (0.69, 0.8)	0.0006 (-0.0002, 0.0015)	0.0036 (-0.059, 0.066)	-0.055 (-0.148, 0.037)	-0.052 (-0.127, 0.023)	0.03897
235	Open Wounds of Head; Neck; and Trunk	9.87 (9.71, 10.03)	0.81 (0.78, 0.84)	0.0008 (0.0003, 0.0013)	-0.2206 (-0.259, -0.182)	-0.023 (-0.079, 0.034)	-0.244 (-0.289, -0.198)	0
236	Open Wounds of Extremities	13.44 (13.25, 13.63)	0.7 (0.66, 0.75)	-0.0002 (-0.0009, 0.0004)	0.0295 (-0.017, 0.076)	0.046 (-0.023, 0.114)	0.075 (0.02, 0.13)	0.00000
237	Complication of Device; Implant or Graft	6.31 (6.18, 6.44)	0.69 (0.64, 0.74)	0.0031 (0.0023, 0.0038)	-0.0039 (-0.06, 0.052)	-0.017 (-0.099, 0.066)	-0.021 (-0.088, 0.046)	0.51705
238	Complications of Surgical Procedures or Medical Care	7.96 (7.81, 8.11)	0.66 (0.6, 0.73)	0.0034 (0.0025, 0.0043)	0.5494 (0.482, 0.617)	0.065 (-0.034, 0.165)	0.614 (0.534, 0.695)	0

© 2020 Ellis RP et al. *JAMA Network Open.*

CCS	Category label	9/15 Rate	α	β	γ	δ	$\gamma + \delta$	F: γ=δ=0
239	Superficial Injury; Contusion	42.34 (42, 42.68)	0.56 (0.5, 0.62)	0.0014 (0.0005, 0.0023)	-0.1091 (-0.175, -0.043)	-0.042 (-0.139, 0.055)	-0.151 (-0.23, -0.073)	0
240	Burns	2.32 (2.24, 2.4)	0.72 (0.67, 0.77)	0.0003 (-0.0004, 0.0011)	0.004 (-0.051, 0.059)	-0.019 (-0.1, 0.062)	-0.015 (-0.08, 0.051)	0.65859
241	Poisoning by Psychotropic Agents	0.34 (0.31, 0.37)	0.76 (0.69, 0.84)	0.0017 (0.0006, 0.0028)	-0.4971 (-0.58, -0.415)	-0.073 (-0.194, 0.049)	-0.57 (-0.668, -0.471)	0
242	Poisoning by Other Medications and Drugs	3.96 (3.86, 4.06)	0.83 (0.78, 0.88)	0.0027 (0.0019, 0.0035)	-0.7927 (-0.853, -0.733)	-0.06 (-0.148, 0.028)	-0.853 (-0.925, -0.782)	0
243	Poisoning by Nonmedicinal Substances	4.22 (4.11, 4.33)	0.26 (0.17, 0.35)	0.0026 (0.0012, 0.0039)	-0.0122 (-0.115, 0.09)	0.001 (-0.149, 0.152)	-0.011 (-0.133, 0.111)	0.87056
244	Other Injuries and Conditions Due to External Causes	33.45 (33.15, 33.75)	0.56 (0.51, 0.6)	0.0038 (0.0031, 0.0045)	0.1857 (0.134, 0.238)	-0.135 (-0.212, -0.059)	0.05 (-0.012, 0.112)	0
245	Syncope	9.62 (9.46, 9.78)	0.77 (0.72, 0.81)	0.0029 (0.0023, 0.0036)	-0.016 (-0.066, 0.034)	-0.043 (-0.117, 0.031)	-0.059 (-0.119, 0.001)	0.00156
246	Fever of Unknown Origin	30.41 (30.12, 30.7)	0.98 (0.62, 1.35)	0.009 (0.0036, 0.0144)	-0.1207 (-0.522, 0.281)	0.036 (-0.555, 0.626)	-0.085 (-0.564, 0.394)	0.46828
247	Lymphadenitis	9.34 (9.18, 9.5)	0.79 (0.73, 0.86)	0.0037 (0.0027, 0.0046)	0.0075 (-0.065, 0.08)	-0.094 (-0.201, 0.012)	-0.087 (-0.173, 0)	0.00132
248	Gangrene	0.23 (0.21, 0.25)	0.87 (0.78, 0.96)	0.0033 (0.002, 0.0046)	0.023 (-0.076, 0.122)	0.120 (-0.025, 0.265)	0.143 (0.025, 0.261)	0.00011
249	Shock	0.47 (0.43, 0.51)	0.65 (0.58, 0.72)	0.0056 (0.0045, 0.0066)	-0.0442 (-0.12, 0.031)	-0.017 (-0.128, 0.094)	-0.061 (-0.151, 0.029)	0.0089
250	Nausea and Vomiting	35.9 (35.59, 36.21)	0.61 (0.54, 0.68)	0.0072 (0.0062, 0.0083)	0.0054 (-0.074, 0.085)	-0.104 (-0.221, 0.013)	-0.098 (-0.193, -0.003)	0.00096
251	Abdominal Pain	81.23 (80.76, 81.7)	0.8 (0.75, 0.85)	0.0025 (0.0018, 0.0033)	0.1314 (0.075, 0.188)	-0.045 (-0.128, 0.039)	0.087 (0.019, 0.154)	0
252	Malaise and Fatigue	57.86 (57.46, 58.26)	0.58 (0.51, 0.64)	0.0052 (0.0043, 0.0061)	-0.0274 (-0.096, 0.041)	-0.069 (-0.17, 0.033)	-0.096 (-0.178, -0.014)	0.00014
253	Allergic Reactions	95.25 (94.74, 95.76)	0.61 (0.56, 0.67)	0.0038 (0.0029, 0.0046)	-0.0138 (-0.076, 0.048)	-0.030 (-0.12, 0.061)	-0.043 (-0.117, 0.03)	0.08363
254	Rehabilitation Care; Fitting of Prostheses; and Adjustment of Devices	21.07 (20.83, 21.31)	0.68 (0.63, 0.73)	0.0032 (0.0024, 0.004)	-0.9133 (-0.973, -0.854)	-0.079 (-0.166, 0.009)	-0.992 (-1.063, -0.921)	0

© 2020 Ellis RP et al. *JAMA Network Open.*

CCS	Category label	9/15 Rate	α	β	γ	δ	$\gamma + \delta$	F: γ=δ=0
255	Administrative/Social Admission	205.14 (204.4, 205.88)	0.69 (0.58, 0.8)	0.0018 (0.0002, 0.0035)	-0.6761 (-0.797, -0.555)	0.018 (-0.159, 0.196)	-0.658 (-0.802, -0.514)	0
256	Medical Examination/Evaluation	296.86 (295.98, 297.74)	0.72 (0.61, 0.82)	0.0027 (0.0012, 0.0043)	0.5352 (0.42, 0.651)	0.061 (-0.109, 0.231)	0.597 (0.459, 0.735)	0
257	Other Aftercare	85.62 (85.14, 86.1)	0.39 (0.32, 0.46)	0.0079 (0.0068, 0.0089)	0.1497 (0.071, 0.229)	0.025 (-0.091, 0.141)	0.175 (0.081, 0.269)	0
258	Other Screening for Suspected Conditions (Not Mental Disorders or Infectious Disease)	103.24 (102.71, 103.77)	0.34 (0.26, 0.42)	0.0076 (0.0064, 0.0088)	0.5338 (0.444, 0.623)	0.174 (0.042, 0.306)	0.708 (0.601, 0.815)	0
259	Residual Codes; Unclassified	192.87 (192.15, 193.59)	0.35 (0.24, 0.46)	0.0079 (0.0062, 0.0096)	0.2882 (0.163, 0.414)	0.06 (-0.124, 0.245)	0.348 (0.199, 0.498)	0
650	Adjustment Disorders	59.3 (58.9, 59.7)	0.92 (0.88, 0.97)	0.001 (0.0003, 0.0016)	-0.0858 (-0.134, -0.037)	0.209 (0.138, 0.28)	0.123 (0.065, 0.181)	0
651	Anxiety Disorders	162.86 (162.2, 163.52)	0.42 (0.37, 0.47)	0.0077 (0.007, 0.0084)	0.02 (-0.033, 0.073)	0.056 (-0.022, 0.133)	0.076 (0.013, 0.138)	0.0001
652	Attention-Deficit Conduct and Disruptive Behavior Disorders	78.56 (78.1, 79.02)	0.5 (0.45, 0.55)	0.007 (0.0063, 0.0077)	-0.0183 (-0.07, 0.034)	-0.153 (-0.23, -0.077)	-0.172 (-0.234, -0.11)	0
653	Delirium Dementia and Amnestic and Other Cognitive Disorders	3.49 (3.39, 3.59)	0.49 (0.43, 0.55)	0.0065 (0.0056, 0.0074)	-0.1984 (-0.264, -0.133)	-0.126 (-0.223, -0.03)	-0.325 (-0.403, -0.246)	0
654	Developmental Disorders	14.18 (13.98, 14.38)	0.43 (0.38, 0.48)	0.0074 (0.0066, 0.0081)	-0.0107 (-0.069, 0.048)	0.066 (-0.02, 0.152)	0.056 (-0.014, 0.125)	0.01025
656	Impulse Control Disorders NEC	1.69 (1.62, 1.76)	0.63 (0.59, 0.67)	0.0055 (0.0049, 0.0061)	-0.073 (-0.118, -0.028)	-0.073 (-0.138, -0.007)	-0.146 (-0.199, -0.092)	0
657	Mood Disorders	165.94 (165.27, 166.61)	0.8 (0.76, 0.84)	0.0026 (0.0021, 0.0032)	-0.0829 (-0.125, -0.041)	0.034 (-0.027, 0.096)	-0.049 (-0.099, 0.001)	0
658	Personality Disorders	2.88 (2.79, 2.97)	0.47 (0.42, 0.53)	0.0065 (0.0057, 0.0073)	-0.1025 (-0.165, -0.04)	-0.086 (-0.178, 0.005)	-0.189 (-0.263, -0.114)	0
659	Schizophrenia and Other Psychotic Disorders	4.38 (4.27, 4.49)	0.73 (0.69, 0.76)	0.004 (0.0034, 0.0045)	-0.1642 (-0.207, -0.122)	-0.073 (-0.136, -0.011)	-0.238 (-0.288, -0.187)	0
660	Alcohol-Related Disorders	11.31 (11.13, 11.49)	0.5 (0.47, 0.53)	0.0062 (0.0058, 0.0067)	-0.0222 (-0.058, 0.013)	0.014 (-0.039, 0.066)	-0.009 (-0.051, 0.034)	0.06622
661	Substance-Related Disorders	15.95 (15.74, 16.16)	0.41 (0.33, 0.49)	0.0085 (0.0073, 0.0097)	1.151 (1.061, 1.241)	0.388 (0.255, 0.52)	1.539 (1.432, 1.646)	0

© 2020 Ellis RP et al. *JAMA Network Open.*

CCS	Category label	9/15 Rate	α	β	γ	δ	$\gamma + \delta$	F: γ=δ=0
662	Suicide and Intentional Self-Inflicted Injury	1.65 (1.58, 1.72)	0.3 (0.22, 0.39)	0.0089 (0.0076, 0.0102)	0.208 (0.113, 0.303)	0.203 (0.063, 0.343)	0.411 (0.297, 0.524)	0
663	Screening and History of Mental Health and Substance Abuse Codes	47.9 (47.54, 48.26)	0.27 (0.2, 0.34)	0.0094 (0.0083, 0.0104)	-0.696 (-0.774, -0.618)	-0.147 (-0.262, -0.032)	-0.843 (-0.937, -0.75)	0
670	Miscellaneous Mental Health Disorders	21.87 (21.63, 22.11)	0.52 (0.46, 0.57)	0.0054 (0.0046, 0.0062)	-0.0911 (-0.151, -0.032)	0.123 (0.035, 0.211)	0.032 (-0.039, 0.103)	0
2601	External Cause Codes: Cut/Pierce	2.04 (1.97, 2.11)	0.36 (0.27, 0.46)	0.0056 (0.0042, 0.007)	-0.0092 (-0.113, 0.095)	-0.148 (-0.301, 0.005)	-0.157 (-0.281, -0.033)	0.00006
2602	External Cause Codes: Drowning/Submersion	0.01 (0, 0.02)	-0.05 (-0.53, 0.43)	0.0066 (-0.0005, 0.0137)	0.209 (-0.322, 0.739)	-0.974 (-1.754, -0.194)	-0.765 (-1.398, -0.133)	0.00003
2603	External Cause Codes: Fall	7.95 (7.8, 8.1)	0.47 (0.36, 0.58)	0.007 (0.0054, 0.0087)	0.1889 (0.065, 0.312)	-0.281 (-0.462, -0.099)	-0.092 (-0.239, 0.056)	0
2604	External Cause Codes: Fire/Burn	0.32 (0.29, 0.35)	0.34 (0.23, 0.45)	0.0067 (0.0051, 0.0083)	0.074 (-0.046, 0.194)	-0.305 (-0.482, -0.128)	-0.231 (-0.374, -0.088)	0
2605	External Cause Codes: Firearm	0.07 (0.06, 0.08)	0.3 (0.18, 0.43)	0.0096 (0.0077, 0.0114)	0.219 (0.083, 0.355)	0.022 (-0.178, 0.223)	0.241 (0.079, 0.404)	0
2606	External Cause Codes: Machinery	0.09 (0.07, 0.11)	0.32 (0.17, 0.48)	0.0052 (0.003, 0.0075)	0.732 (0.565, 0.9)	-0.643 (-0.89, -0.396)	0.089 (-0.111, 0.289)	0
2607	External Cause Codes: Motor Vehicle Traffic (MVT)	3.51 (3.41, 3.61)	0.28 (0.21, 0.35)	0.0074 (0.0064, 0.0084)	0.136 (0.059, 0.212)	-0.081 (-0.194, 0.031)	0.054 (-0.037, 0.145)	0
2608	External Cause Codes: Pedal Cyclist; Not MVT	0.48 (0.44, 0.52)	0.04 (-0.08, 0.15)	0.0043 (0.0025, 0.006)	-0.0778 (-0.207, 0.051)	-0.094 (-0.284, 0.097)	-0.171 (-0.325, -0.017)	0.00012
2609	External Cause Codes: Pedestrian; Not MVT	0.03 (0.02, 0.04)	0.23 (-0.01, 0.46)	0.0038 (0.0003, 0.0073)	0.804 (0.545, 1.064)	-0.107 (-0.489, 0.275)	0.697 (0.388, 1.007)	0
2610	External Cause Codes: Transport; Not MVT	0.57 (0.53, 0.61)	0.06 (-0.34, 0.47)	0.0052 (-0.0008, 0.0113)	1.132 (0.682, 1.581)	-1.244 (-1.905, -0.583)	-0.112 (-0.648, 0.424)	0
2611	External Cause Codes: Natural/Environment	4.87 (4.75, 4.99)	-0.19 (-0.49, 0.11)	0.0091 (0.0047, 0.0136)	0.906 (0.574, 1.237)	0.518 (0.031, 1.006)	1.424 (1.029, 1.819)	0
2612	External Cause Codes: Overexertion	2.31 (2.23, 2.39)	0.47 (0.36, 0.59)	0.0065 (0.0048, 0.0082)	-0.823 (-0.949, -0.696)	-0.235 (-0.421, -0.049)	-1.058 (-1.209, -0.907)	0

© 2020 Ellis RP et al. *JAMA Network Open.*

CCS	Category label	9/15 Rate	α	β	γ	δ	$\gamma + \delta$	F: γ=δ=0
2613	External Cause Codes: Poisoning	0.62 (0.58, 0.66)	0.19 (0.04, 0.34)	0.0087 (0.0066, 0.0107)	-0.770 (-1.023, -0.517)	-0.497 (-1.993, 0.999)	-1.267 (-2.572, 0.039)	0
2614	External Cause Codes: Struck by; Against	4.51 (4.4, 4.62)	0.32 (0.24, 0.41)	0.0049 (0.0036, 0.0062)	0.133 (0.038, 0.227)	-0.155 (-0.293, -0.016)	-0.022 (-0.134, 0.091)	0
2616	Adverse Effects of Medical Care	0.4 (0.37, 0.43)	0.43 (0.3, 0.56)	0.0074 (0.0055, 0.0093)	-0.244 (-0.386, -0.102)	-0.408 (-0.617, -0.199)	-0.653 (-0.822, -0.483)	0
2617	Adverse Effects of Medical Drugs	1.33 (1.27, 1.39)	0.39 (0.27, 0.51)	0.0066 (0.0048, 0.0084)	1.606 (1.47, 1.742)	0.232 (0.031, 0.432)	1.837 (1.675, 2)	0
2618	External Cause Codes: Other Specified and Classifiable	1.54 (1.48, 1.6)	0.28 (0.17, 0.39)	0.0065 (0.0049, 0.0081)	-0.328 (-0.445, -0.211)	-0.182 (-0.355, -0.01)	-0.511 (-0.65, -0.371)	0
2619	External Cause Codes: Other Specified; NEC	1.54 (1.48, 1.6)	0.09 (-0.03, 0.21)	0.0086 (0.0069, 0.0104)	-0.656 (-0.787, -0.526)	-0.297 (-0.489, -0.105)	-0.953 (-1.109, -0.797)	0
2620	External Cause Codes: Unspecified	6.54 (6.41, 6.67)	0.39 (0.29, 0.49)	0.0044 (0.0029, 0.0058)	0.519 (0.41, 0.628)	-0.398 (-0.558, -0.237)	0.121 (-0.009, 0.252)	0
2621	External Cause Codes: Place of Occurrence	7.29 (7.15, 7.43)	0.38 (0.3, 0.47)	0.0049 (0.0036, 0.0061)	0.161 (0.067, 0.255)	-0.319 (-0.457, -0.181)	-0.158 (-0.27, -0.046)	0
ALL	# of categories (out of 282), where coefficients are statistically significant (that is, P < 0.05/282)		275	249	107	77	145	164
ALL	# of categories with coefficients that are statistically significant and large (that is, ≥±20%)		-	-	46	27	61	-

© 2020 Ellis RP et al. *JAMA Network Open.*
